# A Hybrid-Level Weighted Multi-View Measurement Method for Industrial Geometric Features

**DOI:** 10.3390/s26216834

**Published:** 2026-10-29

**Authors:** Fangyuan Wang, Jibin Zhao

**Affiliations:** 1 State Key Laboratory of Robotics and Intelligent System, Shenyang Institute of Automation, Chinese Academy of Sciences, Shenyang 110169, China; wangfangyuan@sia.cn; 2 University of Chinese Academy of Sciences, Beijing 100049, China

**Keywords:** multi-view geometry, industrial measurement, three-dimensional reconstruction, subpixel edge localization

## Abstract

Multi-view measurement of industrial geometric features is susceptible to reduced three-dimensional (3D) reconstruction accuracy due to inconsistent imaging quality, local edge localization errors, and unfavorable observation poses. To address these issues, this paper proposes a hybrid-level weighted multi-view measurement method that evaluates the reliability of cross-view observations from two complementary aspects: local edge quality and global observation geometry. At the local level, a subpixel grayscale model is established along the edge-normal direction to derive a local quality weight. At the global level, an observation-geometry weight is defined based on the angle between the feature-plane normal and the camera optical axis. The two weights are then hierarchically normalized and multiplicatively fused for each set of cross-view correspondences. The resulting fused weights are incorporated into multi-view 3D reconstruction, thereby reducing the propagation of errors arising from low-quality local observations and unfavorable viewpoints to the reconstruction. Experiments show that, compared with equal-weight reconstruction, the proposed method reduces the mean error in center-to-center distance measurement by 41.5%, and the mean errors in square-hole width and height measurement by 22.8% and 25.8%, respectively. These results demonstrate the high precision, improved stability, and strong extensibility of the proposed method for industrial planar geometric feature measurement.

## 1. Introduction

In recent years, with the continuously increasing requirements of industrial manufacturing for the dimensional inspection accuracy and efficiency of complex parts, non-contact vision-based measurement methods have attracted extensive attention in industrial metrology [1,2,3,4,5]. Among them, multi-view stereo vision can acquire multi-view information of a target from different positions and directions using multiple cameras, offering advantages in expanding spatial coverage, enhancing observability under occlusion, and providing redundant geometric constraints. It has therefore been increasingly applied to 3D measurement and geometric-feature inspection of complex parts. A typical multi-view vision measurement system consists of multiple cameras arranged around the measurement region, and the number of cameras can be extended to more than ten, or even more, according to the workpiece size and measurement range [6,7]. Compared with conventional binocular vision methods [8,9], multi-view stereo vision can exploit information from more viewpoints to strengthen 3D reconstruction constraints, improve reconstruction stability, and enlarge the effective measurement range [10,11,12]. Compared with active non-contact measurement methods such as structured light [13,14], multi-view stereo vision requires no additional projection coding; instead, it can directly utilize the intrinsic geometric edge information of the part and reconstruct features through redundant multi-view constraints. Consequently, multi-view vision is highly applicable to industrial measurement tasks involving distinct geometric features and requiring large spatial coverage.

In multi-view 3D measurement, a spatial point is generally reconstructed jointly from its corresponding two-dimensional (2D) observations across multiple images. Conventional multi-view 3D reconstruction methods typically assume that corresponding observations from different views contribute equally to the estimation of a spatial point, and therefore adopt an equal-weighting strategy for parameter estimation [15]. In practical industrial measurement scenarios, however, cameras differ in installation position, viewing angle, working distance, and local imaging conditions, resulting in nonuniform localization accuracy and reliability of the corresponding 2D feature points. This issue is particularly pronounced for industrial parts with specular surfaces, machined chamfers, and through-holes of finite thickness, where edge sharpness, feature-point localization reliability, and the degree of projective distortion vary across local regions and viewpoints [16,17,18]. If an equal-weight reconstruction strategy is still adopted, low-quality observations contribute as much as high-quality ones, causing localization errors of the feature edges to propagate further into 3D reconstruction and geometric-parameter estimation, thereby reducing measurement accuracy and stability [19,20]. Therefore, appropriately assigning reconstruction weights according to the reliability of different observations is a key issue in improving the accuracy and robustness of multi-view stereo measurement in complex industrial environments.

To address the above issues, previous studies have investigated weighted reconstruction methods from the perspectives of feature imaging quality, observation geometry, and camera structural errors. Bi et al. [21] proposed a weighting model using camera calibration errors and structural-parameter errors to reduce the influence of cameras with relatively large errors on reconstruction results. However, such methods primarily assign weights at the camera level and therefore cannot adequately capture variations in local imaging quality among different edge points within the same view. Moreover, reconstruction-error estimation based on camera structural parameters generally requires pairwise analysis of cameras in a multi-camera system, leading to increased computational complexity as the number of cameras grows [22]. For circular features, Wu et al. [23] used the ratio of the major to minor axes of the projected ellipse to characterize the degree of projective distortion and incorporated it as an important weighting factor affecting edge extraction and circle-center fitting accuracy. Wu et al. [24] further extended this feature-level geometric information to square-hole features and constructed a weighting model based on the geometric relationships between projected edges under perspective projection. These methods can, to some extent, evaluate observation quality by considering feature imaging quality and observation geometry. However, their weighting strategies generally rely on shape-specific constraints of particular geometric features, such as ellipse projection parameters for circular features or edge geometric relationships for square features. Consequently, their applicability is limited when dealing with the wide variety of geometric features encountered in industrial measurement scenarios.

Beyond geometric measurement, the integration of complementary information at multiple levels or across different domains has also been investigated in other computer vision tasks. Liu et al. [25] jointly modeled global semantic similarity and local structural similarity for thermal infrared object tracking, while Yuan et al. [26] integrated complementary spatial- and frequency-domain information for cross-modal image fusion. These studies demonstrate the effectiveness of combining complementary information from different levels or domains. In contrast, the present work does not learn latent feature similarities or cross-modal representations. Instead, it constructs physically interpretable reliability measures from point-level local edge quality and view-level global observation geometry, and directly incorporates the resulting weights into multi-view 3D reconstruction.

To overcome the limitations of existing weighted multi-view stereo measurement methods in local edge quality evaluation and feature applicability, this paper proposes a hybrid-level weighted multi-view measurement method for industrial geometric features. The proposed method evaluates observation reliability at two complementary levels: point-level local quality and view-level global observation geometry. First, grayscale information is extracted from the neighborhood along the normal direction of the target feature edge, and a subpixel edge model is established to simultaneously obtain a subpixel estimate of the edge position and evaluate local imaging quality and localization reliability. Second, a global observation weight is constructed based on the geometric relationship between the normal vector of the target feature plane and the camera optical axis, thereby characterizing the observation geometry under different viewpoints and its influence on reconstruction accuracy. Finally, each set of cross-view corresponding points is treated as the basic unit for reconstruction. The local quality weights and global observation-geometry weights are hierarchically normalized and multiplicatively fused, and the resulting fused weights are incorporated into multi-view 3D reconstruction to reduce the propagation of errors arising from low-quality edge observations and unfavorable viewpoints.

The main contributions of this paper are summarized as follows:

(1) A local edge quality evaluation and weighting method based on grayscale distribution modeling in the edge neighborhood is proposed. A subpixel edge model is established to obtain subpixel estimates of edge positions, while edge contrast, grayscale transition width, and model-fitting consistency are jointly used to evaluate the reliability of edge points. This strategy reduces the influence of low-quality local observations on 3D reconstruction.
(2) A view-level weighting method based on global feature observation geometry is proposed. The geometric relationship between the target feature and the camera is used to characterize the quality of observation geometry from different viewpoints. This weighting strategy reduces the contribution of unfavorable observation poses, such as large viewing angles, to the reconstruction results, thereby improving the stability and robustness of multi-view stereo measurement.
(3) A hybrid-level weighted measurement framework integrating local edge quality and global observation geometry is established. The framework does not explicitly rely on shape-specific projection constraints of a particular geometric feature. It can therefore be applied to the measurement of typical industrial planar geometric features, such as circular and square holes, and has the potential to be further extended to other measurement tasks based on edge-point reconstruction.

The remainder of this paper is organized as follows. Section 2 introduces the fundamental principles of multi-view 3D reconstruction and weighted least squares, providing the theoretical basis for the proposed method. Section 3 presents the proposed hybrid-level weighted multi-view measurement method in detail, including the local edge weighting strategy, the global observation-geometry weighting strategy, and the hybrid-level weight fusion strategy. Section 4 describes the experimental platform, experimental design, and result analysis, and evaluates the proposed method in terms of measurement accuracy, robustness to image noise, and extensibility to different geometric features. Finally, the paper concludes with a summary of the present work and a discussion of directions for future research.

## 2. Multi-View 3D Reconstruction

### 2.1. Reconstruction Principle

**Figure 1:**
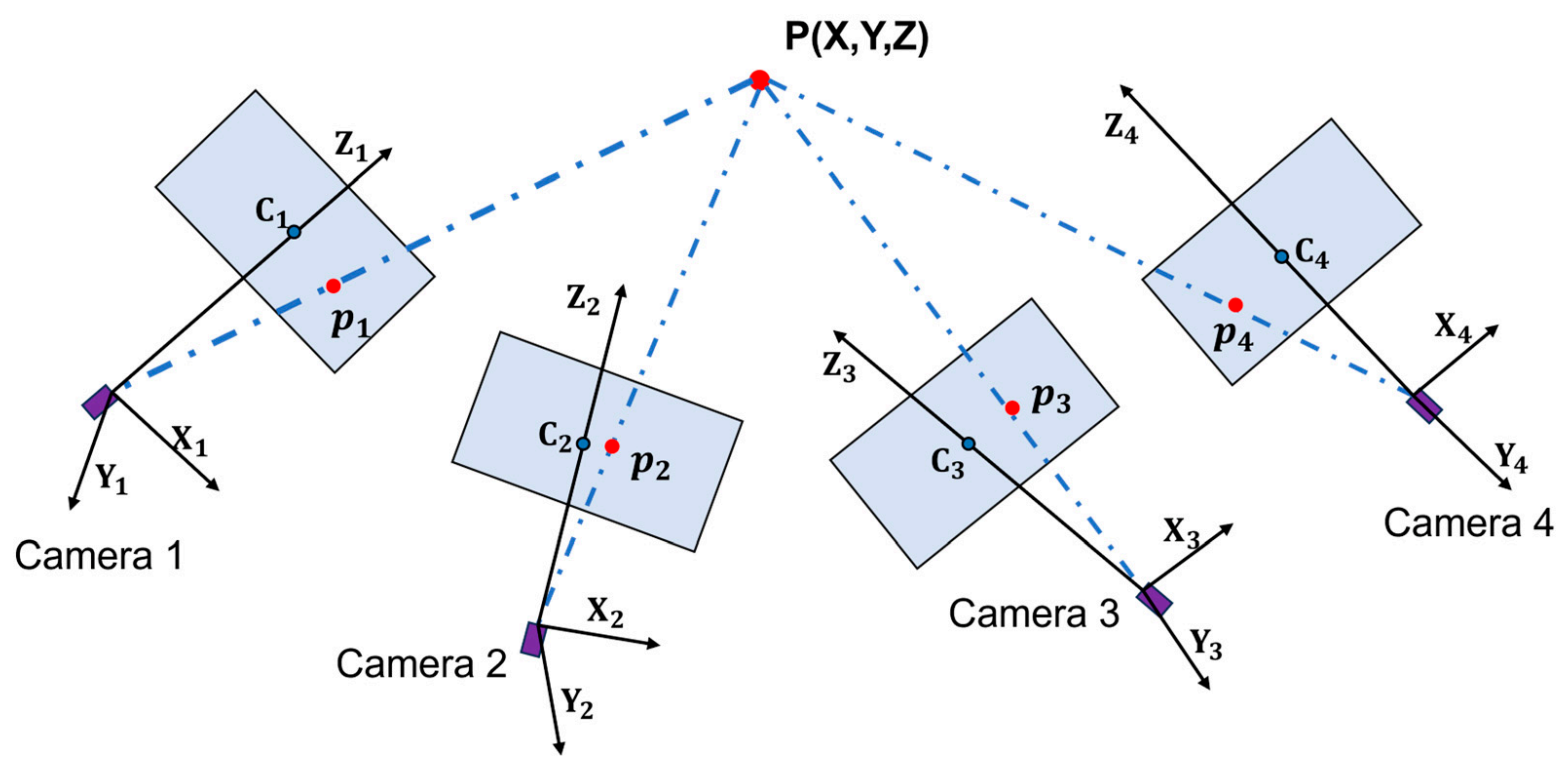
Ideal multi-view 3D reconstruction model. The blue points $C_{i}$ denote the principal points, while the red points $p_{i}$ denote the projections of the spatial point P in different views. The dashed lines represent projection rays passing through the corresponding image points and the solid lines indicate the camera coordinate system.

Conventional multi-view 3D reconstruction relies on observations of the same spatial point from multiple cameras. Based on the pinhole camera model and the calibrated camera parameters, the projection equations of the spatial point in multiple cameras are jointly formulated to estimate its 3D coordinates. Ideally, as illustrated in Figure 1, multiple projection rays originating from the camera centers and passing through the corresponding feature points $p_{i}$ on the image planes intersect at a unique spatial point P.

According to the pinhole camera model, the projection of a 3D point P onto the image plane of camera i can be expressed as:

(1) $$\lambda_{i}\left[\begin{array}{c} u_{i}\\ v_{i}\\ 1 \end{array}\right]=\left[\begin{array}{ccc} m_{00}^{i} & m_{01}^{i} & \begin{array}{cc} m_{02}^{i} & m_{03}^{i} \end{array}\\ m_{10}^{i} & m_{11}^{i} & \begin{array}{cc} m_{12}^{i} & m_{13}^{i} \end{array}\\ m_{20}^{i} & m_{21}^{i} & \begin{array}{cc} m_{22}^{i} & m_{23}^{i} \end{array} \end{array}\right]\left[\begin{array}{c} \begin{array}{c} X\\ Y \end{array}\\ Z\\ 1 \end{array}\right]=P_{i}\left[\begin{array}{c} X\\ Y\\ \begin{array}{c} Z\\ 1 \end{array} \end{array}\right],$$

where the subscript i indicates that the corresponding parameters are associated with camera i, and this notation is omitted from further explanation hereafter. The matrix P denotes the camera projection matrix, which is determined from the intrinsic and extrinsic camera parameters; (u, v) denotes the coordinates of the projected point in the image coordinate system; (X, Y, Z) denotes the coordinates of the spatial point in the reference coordinate system; and the remaining scalar term λ denotes the homogeneous scale factor. By eliminating the homogeneous scale factor, the following equations are obtained:

(2) $$\left(m_{20}^{i}u_{i}-m_{00}^{i}\right)X+\left(m_{21}^{i}u_{i}-m_{01}^{i}\right)Y+\left(m_{22}^{i}u_{i}-m_{02}^{i}\right)Z=m_{03}^{i}-m_{23}^{i}u_{i},$$

(3) $$\left(m_{20}^{i}v_{i}-m_{10}^{i}\right)X+\left(m_{21}^{i}v_{i}-m_{11}^{i}\right)Y+\left(m_{22}^{i}v_{i}-m_{12}^{i}\right)Z=m_{13}^{i}-m_{23}^{i}v_{i},$$

For a spatial point within the field of view, each camera provides two projection equations. When the point is simultaneously observed by n cameras, Equations (2) and (3) can be extended as follows:

(4) $$\left[\begin{array}{c} \begin{array}{ccc} m_{20}^{1}u_{1}-m_{00}^{1} & m_{21}^{1}u_{1}-m_{01}^{1} & m_{22}^{1}u_{1}-m_{02}^{1} \end{array}\\ \begin{array}{ccc} \;\;\vdots \;\; & \;\;\vdots \;\; & \;\;\vdots \;\; \end{array}\\ \begin{array}{ccc} m_{20}^{n}v_{n}-m_{10}^{n} & m_{21}^{n}v_{n}-m_{11}^{n} & m_{22}^{n}v_{n}-m_{12}^{n} \end{array} \end{array}\right]\left[\begin{array}{c} X\\ Y\\ Z \end{array}\right]=\left[\begin{array}{c} m_{03}^{1}-m_{23}^{1}u_{1}\\ \vdots \\ m_{13}^{n}-m_{23}^{n}v_{n} \end{array}\right],$$

The above equations can be rewritten in matrix form as:

(5) $$AX_{w}=B,$$

In practical measurement environments, due to the effects of image noise, lens distortion, and accumulated numerical errors, the system generally does not admit an exact solution. Geometrically, this means that the projection rays corresponding to the same spatial point do not intersect at a single point. Therefore, the coordinates of the spatial point $X_{w}$ in the reference coordinate system are typically estimated using the least-squares method:

(6) $$X_{w}={\left(A^{T}A\right)}^{-1}A^{T}B,$$

### 2.2. Weighted Reconstruction Principle

In practical industry measurement, the quality of 2D observations of the same spatial point generally varies across different views [18]. Therefore, unlike ordinary least squares, which assigns equal contributions to all observations, weighted reconstruction assigns different weights to the projection equations associated with individual 2D observations. In this way, observations with higher reliability make greater contributions to the estimation of the spatial point, whereas less reliable observations are suppressed. The weighted least-squares formulation can be expressed as:

(7) $$X_{w}={\left(A^{T}WA\right)}^{-1}A^{T}WB,$$

where W denotes a diagonal weight matrix. Since each 2D observation provides two coupled projection constraints derived from the same image observation, the same scalar weight is assigned to the two corresponding equations to regulate the overall contribution of that observation to 3D reconstruction. In the proposed method, the local reliability information is obtained from the grayscale transition along the edge-normal direction. Therefore, it primarily characterizes the stability of edge localization in the normal direction, whereas localization uncertainty in the tangential direction is not independently estimated. A rigorous anisotropic treatment would require constructing a two-dimensional covariance model in the local normal–tangential coordinate system and propagating the corresponding uncertainty into the reconstruction equations. Since the present one-dimensional grayscale model does not provide an independent estimate of tangential uncertainty, a scalar observation weight is adopted as a practical approximation. Extension to a full anisotropic covariance formulation will be considered in future work.

It should be emphasized that this scalar weight is used as an observation-level reliability coefficient. The key innovation of this work lies in appropriately evaluating the reliability of each cross-view corresponding observation and mapping this reliability to suitable reconstruction weights, thereby controlling error propagation and improving reconstruction accuracy and robustness.

## 3. Hybrid-Level Weighted Multi-View Vision Measurement Method

In multi-view stereo measurement, three-dimensional reconstruction errors mainly arise from two sources: localization errors of key feature edge points in the images, and feature projection deformation caused by the geometric relationship between the camera observation pose and the measured object [16,27]. Existing weighted reconstruction methods generally focus on evaluating observation quality based on the overall projection deformation of the target feature in the two-dimensional image. However, such global indicators cannot fully characterize the differences among individual edge points of the same feature in terms of local grayscale distribution, edge blur, specular reflection, and noise disturbance. When local edge points with low localization reliability contribute equally to corresponding-point reconstruction together with high-quality observations, their errors can further propagate into 3D point estimation and geometric parameter estimation, thereby degrading measurement accuracy and stability.

**Figure 2:**
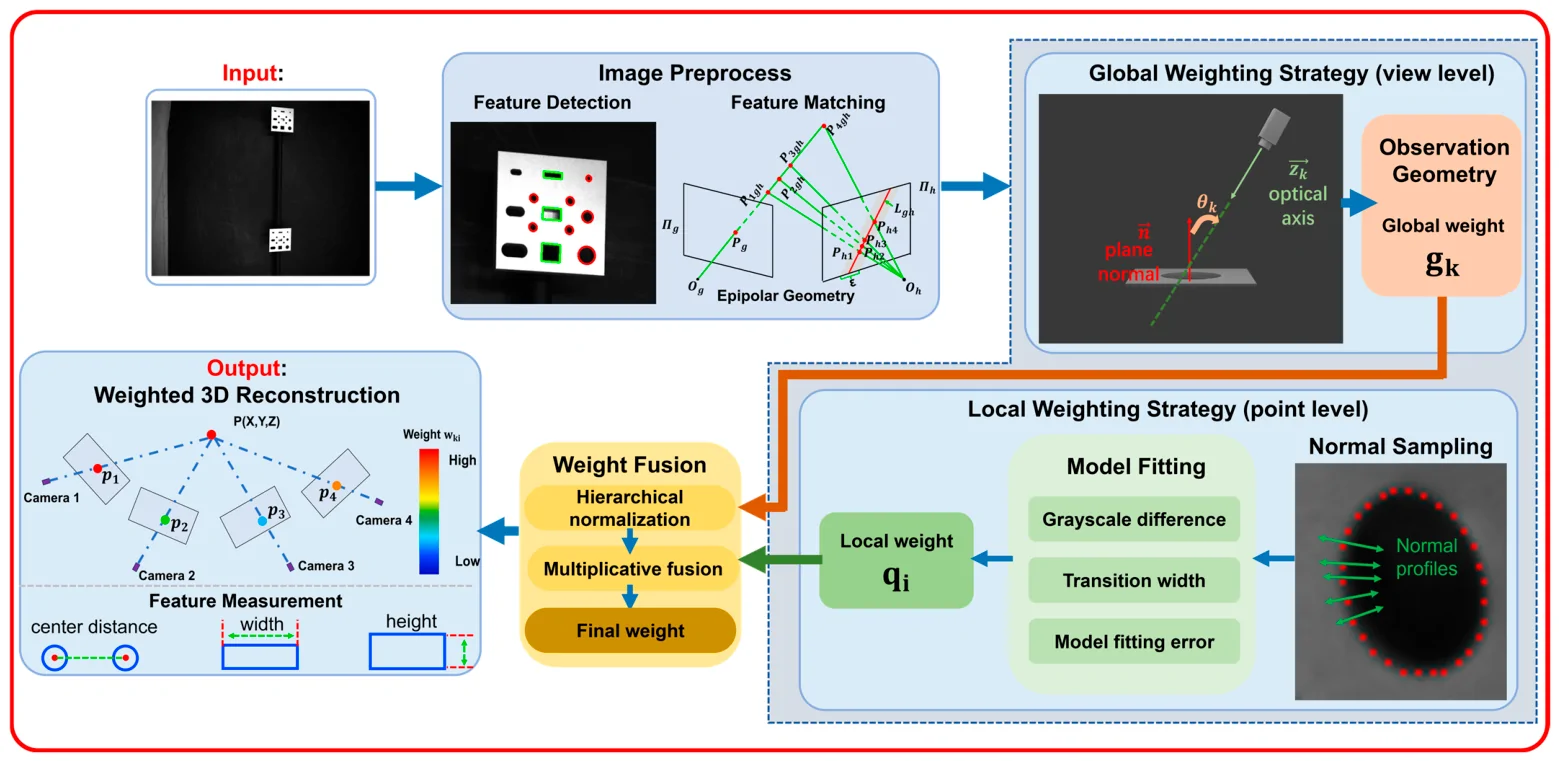
Overall workflow of the proposed hybrid-level weighted multi-view measurement method.

To address these issues, this paper proposes a hybrid-level weighted multi-view measurement method for industrial geometric features, as illustrated in the overall workflow in Figure 2. First, during image preprocessing, synchronously acquired multi-camera images are used as input. The initial feature boundaries are extracted using the conventional Canny edge detector [28], providing pixel-level edge candidates for subsequent processing. Then cross-view feature correspondences are established under epipolar geometric constraints, providing the data basis for the joint reconstruction of corresponding edge points. During correspondence filtering, an epipolar-distance threshold of 1.5 pixels is adopted and this value is empirically selected in the present experiments to balance matching robustness and correspondence accuracy. Since the conventional initial edge-detection and cross-view matching procedures are not the focus of this work, only their main implementation steps are briefly described here; further details can be found in [15,27,28,29]. On this basis, observation reliability is evaluated separately at the levels of local edge quality and global observation geometry, and the two are then fused to obtain the weights used for multi-view 3D reconstruction. The following subsections describe the local edge weighting strategy, the global observation-geometry weighting strategy, and the hybrid-level weight fusion method, respectively.

### 3.1. Local Edge Weighting Strategy

**Figure 3:**
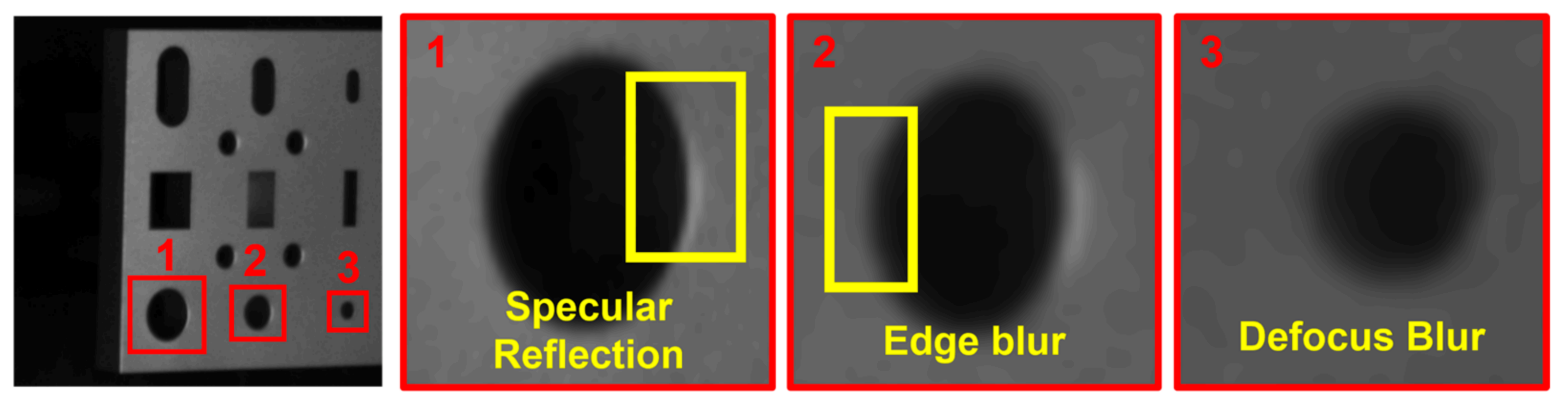
Example of edge blurring in an image acquired under practical industrial measurement conditions.

Localization errors of 2D edge points constitute one of the major sources of error affecting the accuracy of multi-view 3D reconstruction. Under ideal imaging conditions, a target edge can be approximated as a step change in grayscale intensity, with a well-defined geometric contour and an unambiguous boundary position. In practical industrial measurement scenarios, however, factors such as nonuniform illumination, surface reflection, machined chamfers, and lens defocus often cause target edges to appear as grayscale transition regions with finite widths, as shown in Figure 3. A broadened edge transition reduces the local image gradient and localization stability, allowing 2D localization errors to further propagate into subsequent feature fitting and 3D reconstruction, ultimately degrading measurement accuracy.

**Figure 4:**
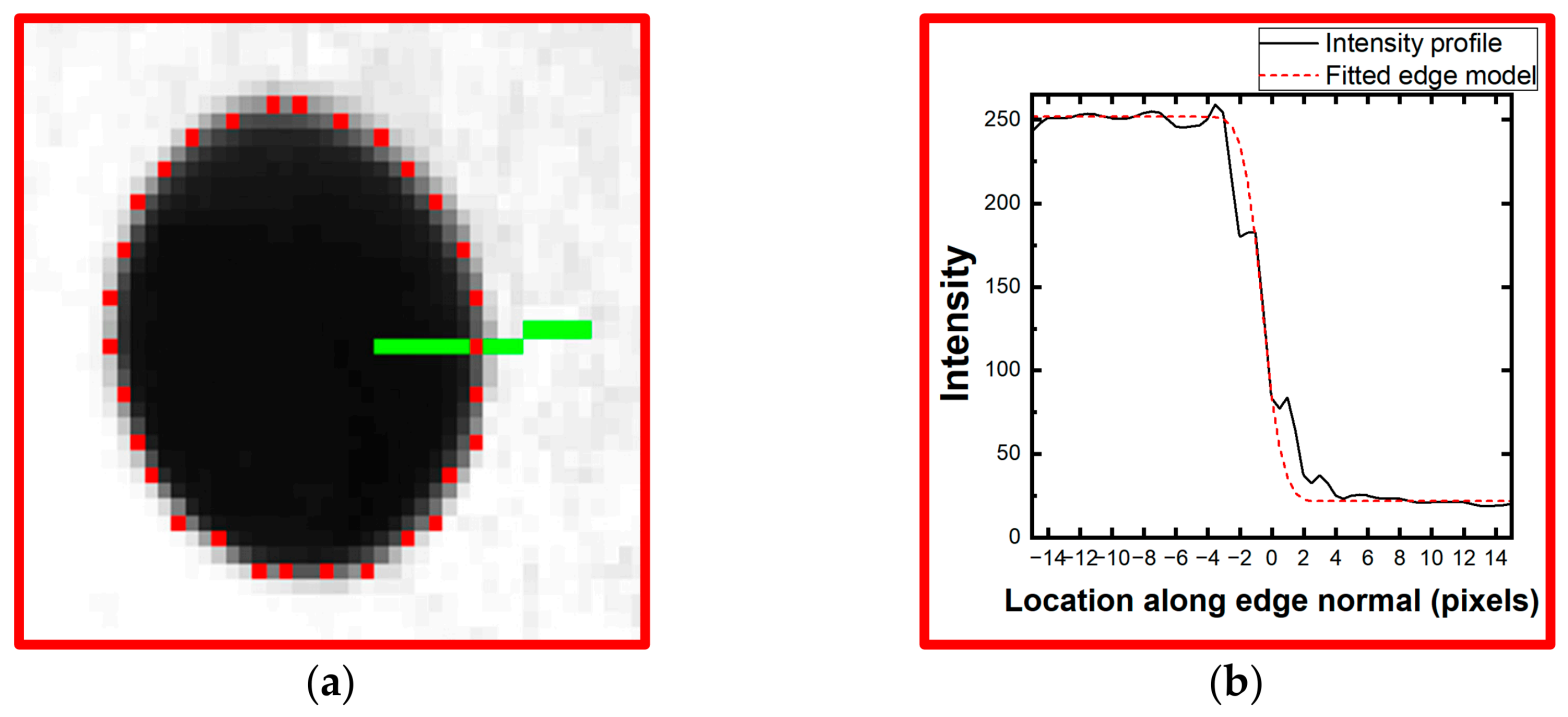
Edge-normal grayscale sampling and subpixel edge model fitting: (**a**) illustration of grayscale sampling along the edge-normal direction; (**b**) grayscale intensity profile along the normal direction and the fitted subpixel edge model.

To quantitatively characterize the local imaging properties of an edge, a local grayscale distribution model is established along the edge-normal direction, from which the subpixel edge position and local quality parameters are estimated. After the target feature edges are obtained during preprocessing, sampling points are uniformly selected along the feature boundary, and a grayscale profile is extracted along the normal direction at each sampling point. Specifically, the radius of the circumscribed circle of the target feature is used as the sampling length, with a sampling interval of 0.5 pixels, as illustrated in Figure 4a, where the red points denote the edge sampling points and the green line segments indicate the corresponding normal directions. Examination of the sampled edge profiles shows that the grayscale intensity along the normal direction generally undergoes a smooth transition from bright to dark or from dark to bright, as shown in Figure 4b. The width of the grayscale transition region reflects the degree of local edge blur and is closely related to edge localization reliability. Therefore, the grayscale profile is modeled using an error function:

(8) $$f\left(x\right)=I_{0}+\frac{A}{2}\times \left(1+erf\left(\frac{x-x_{0}}{\sqrt{2}\times \sigma_{s}}\right)\right),$$

where the error function (erf(·)) is evaluated using the Abramowitz–Stegun approximation. The model parameters $I_{0},\;A,\;x_{0}\;\mathrm{and}\;\sigma_{s}$ represent the grayscale baseline on the dark side of the edge, the grayscale difference between the bright and dark sides, the position of the grayscale transition center, and the width parameter of the grayscale transition region, respectively. In particular, $\sigma_{s}$ characterizes the degree of edge blur. As shown in Figure 4b, the proposed model provides a good fit to the grayscale distribution of the actual edge and effectively captures the width of the edge transition region.

To improve the stability and reproducibility of the nonlinear fitting, the model parameters are initialized adaptively from each sampled grayscale profile. For a profile containing n grayscale samples, the number of samples used at each end of the profile is defined as

(9) $$k=max(3,\left\lceil n/6\right\rceil ),$$

The mean grayscale intensities at the two ends of the profile are then calculated as:

(10) $${\bar{I}}_{L}=\frac{1}{k}\sum_{j=0}^{k-1}I_{j},$$

(11) $${\bar{I}}_{R}=\frac{1}{k}\sum_{j=n-k}^{n-1}I_{j},$$

Accordingly, the initial grayscale baseline and grayscale difference are set to

(12) $$I_{0}=\min\left({\bar{I}}_{L},{\bar{I}}_{R}\right),I_{0}\in \left[0,255\right],$$

(13) $$A=\left|{\bar{I}}_{L}-{\bar{I}}_{R}\right|,A\in \left[0,255\right],$$

The initial transition-center position is determined from the maximum adjacent grayscale variation. Specifically,

(14) $$j^{*}=\arg\underset{j}{\max}\left|I_{j+1}-I_{j}\right|,\;j^{*}\in \left[0,n-2\right],$$

(15) $$x_{0}=j^{*}+0.5,$$

and the midpoint between the $j^{*}$-th and ${(j}^{*}+1)$-th sampling positions is used as the initial value $X_{0}$. The initial transition-width parameter is set to

(16) $$\sigma_{s}=1.0,\sigma_{s}>0,$$

These initialization rules are applied consistently to all sampled edge profiles.

Using Equation (8), nonlinear fitting is performed on the sampled grayscale data to simultaneously estimate the parameters $I_{0},A,X_{0}\;and\sigma_{s}$. Among them, $X_{0}$ corresponds to the center of the grayscale transition and can therefore be used as a subpixel estimate of the edge position. Compared with conventional edge localization methods based on pixel-level coordinates, the proposed model fitting approach provides a continuous subpixel estimate of the edge position, thereby effectively improving edge localization accuracy.

More importantly, the fitted parameters can also be used to characterize the imaging quality of the edge point. In particular, $\sigma_{s}$ directly describes the width of the grayscale transition region. A smaller $\sigma_{s}$ indicates that the grayscale variation is concentrated within a narrow region, corresponding to a sharper local edge and more stable edge localization. Conversely, a larger $\sigma_{s}$ indicates that the grayscale transition is distributed over a wider region, suggesting that the edge is more strongly affected by factors such as defocus, specular reflection, or machined chamfers, and therefore has relatively lower localization reliability.

According to Equation (8), the transition width corresponding to a grayscale change from 10% to 90% can be further derived as approximately $2.563*\sigma_{s}$ pixels. When $\sigma_{s}=1$, the corresponding transition width is approximately 2.56 pixels. From the physical interpretation of the edge grayscale model, once the transition width exceeds approximately 2.56 pixels, the grayscale variation is distributed over multiple pixels, resulting in a substantially weakened local gradient and increased sensitivity of subpixel localization to image noise and fitting errors. Therefore, $\sigma_{s}$ is adopted in this study as the threshold for edge-quality discrimination. When $\sigma_{s}$ > 1, the grayscale transition width exceeds approximately 2.56 pixels, indicating a relatively high degree of edge blur. In this case, the local grayscale information is insufficient to accurately determine the center of the grayscale transition. Considering that the two-dimensional feature fitting process exploits the geometric constraints of the entire feature, the edge position optimized from the entire 2D geometric feature fitting is used as the final edge position at that location. When $\sigma_{s}\leq 1$, the edge exhibits relatively high local imaging quality, and the subpixel edge position obtained from the grayscale model fitting is adopted as the final edge point so as to fully exploit the local grayscale information. It should be emphasized that the proposed local strategy involves two distinct but complementary operations. First, the final edge position is adaptively determined according to the local imaging quality, using either the subpixel grayscale transition center or the position refined by the overall 2D feature fitting. Second, the reliability of the resulting edge observation is evaluated and converted into a local quality weight for subsequent multi-view reconstruction. Therefore, the local strategy affects both edge localization and reconstruction weighting.

Since $\sigma_{s}$ alone is insufficient to comprehensively evaluate edge quality, edge contrast and model-fitting consistency are further incorporated to construct the edge-quality metric. The parameter A characterizes the grayscale contrast between the two sides of the edge, with a larger A indicating a stronger edge response. In addition, the root mean square error (RMSE) between the fitted curve and the observed grayscale values is introduced as a measure of fitting consistency to evaluate how well the model describes the actual grayscale distribution. By jointly considering these three factors, the local quality weight of the i-th sampled edge point is defined as:

(17) $$q_{i}=\frac{A_{i}}{\left(\sigma_{si}+\varepsilon \right)\left({RMSE}_{i}+\varepsilon \right)},$$

where $\varepsilon =1*10^{-6}$ is a small positive constant introduced to prevent division by zero in numerical computation. This formulation indicates that an edge point is assigned a higher weight when the edge contrast is stronger, the degree of blur is lower, and the model-fitting error is smaller. Consequently, high-quality edge points make greater contributions to subsequent geometric fitting and 3D reconstruction, whereas low-quality edge points affected by nonuniform illumination, surface reflection, machined chamfers, or lens defocus are adaptively assigned lower weights. In this way, the propagation of local errors is effectively suppressed, thereby improving the overall reconstruction accuracy and stability.

To further clarify the relative sensitivity of the local quality weight to the three factors in Equation (17), considering that $\varepsilon =10^{-6}$ is introduced only for numerical stabilization and is much smaller than $\sigma_{si}$ and ${RMSE}_{i}$ under normal conditions, Equation (17) can be approximated as:

(18) $$q_{i}\approx \frac{A_{i}}{\sigma_{si}*{RMSE}_{i}},$$

For small relative perturbations, the corresponding first-order variation can be expressed as:

(19) $$\frac{∆q_{i}}{q_{i}}\approx \frac{∆A_{i}}{A_{i}}-\frac{∆\sigma_{\mathrm{si}}}{\sigma_{\mathrm{si}}}-\frac{∆{\mathrm{RMSE}}_{i}}{{\mathrm{RMSE}}_{i}},$$

This relation indicates that Equation (17) does not explicitly assign a higher relative sensitivity to any single factor; instead, the resulting local quality weight is jointly determined by their relative variations under the actual imaging conditions. Therefore, a factor exhibiting a larger relative variation will have a greater influence on the resulting weight. It should be noted that this analysis characterizes the sensitivity of the local quality weight rather than that of the final measurement result. Since the local weights are subsequently normalized across views, fused with the global observation-geometry weights, and incorporated into weighted 3D reconstruction, the final measurement sensitivity also depends on the actual data distribution and the subsequent reconstruction process.

### 3.2. Global Observation-Geometry Weighting Strategy

The local edge weight can evaluate the local imaging quality of an individual edge point; however, because it is primarily derived from grayscale information within the edge-normal neighborhood, it cannot adequately reflect the influence of the camera observation pose on the overall feature projection and reconstruction reliability. Therefore, in addition to local quality weighting, a view-level weight based on global observation geometry is further introduced to characterize the overall observation quality of the feature from different camera viewpoints.

For planar geometric features such as circular and square holes, observation quality depends not only on the local imaging condition of the edges but also on the relative pose between the feature plane and the camera. When the camera optical axis is approximately parallel to the normal vector of the feature plane, the target is observed close to a frontoparallel configuration, and its projected shape largely preserves the original geometry. As the viewing angle increases, the target is increasingly observed under an oblique view, resulting in stronger perspective foreshortening and projective distortion; consequently, the influence of edge localization errors on the 3D reconstruction may also increase. Therefore, it is necessary to evaluate the reliability of observations from different cameras at the view level based on their observation geometry.

**Figure 5:**
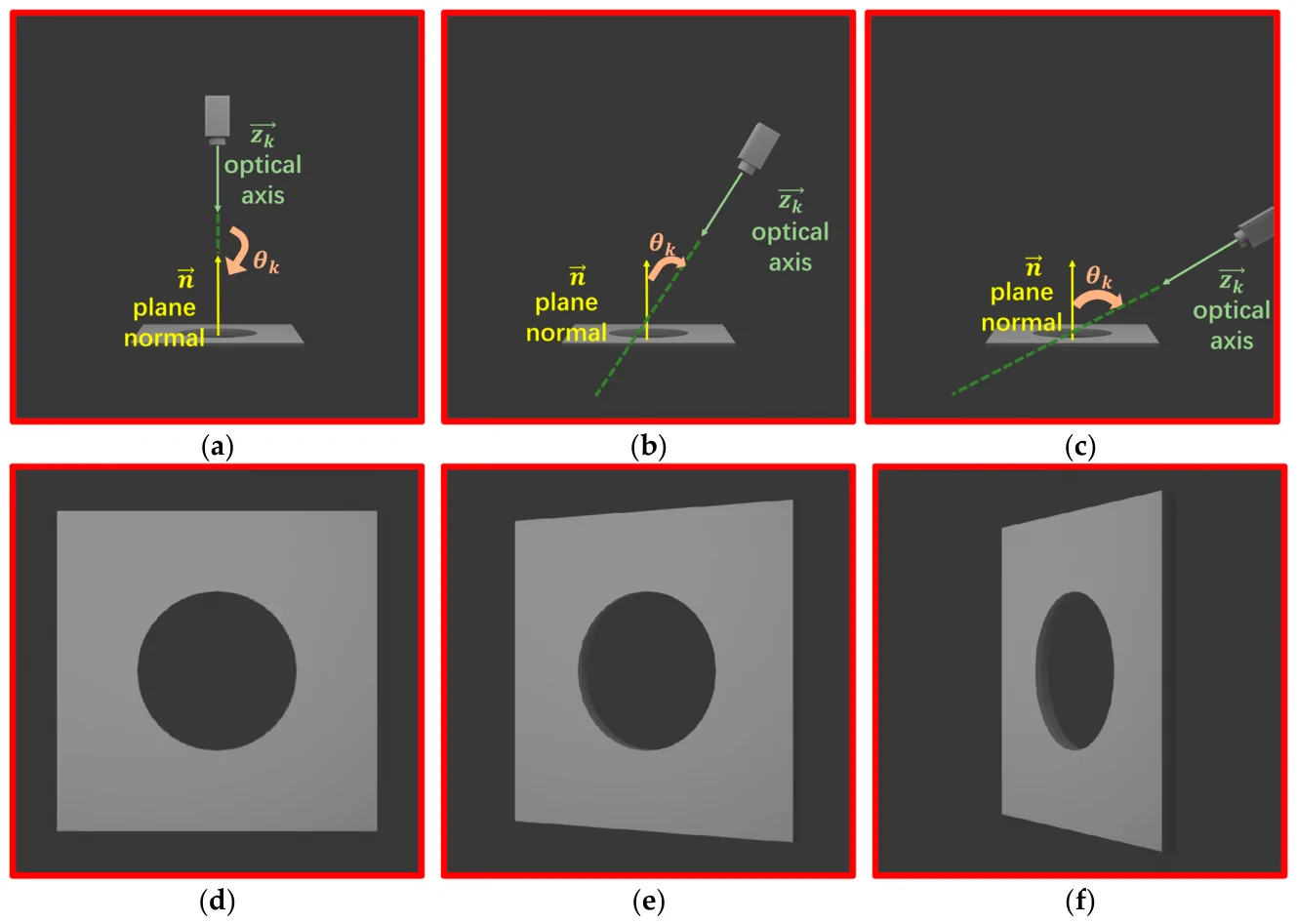
Illustration of the relationship between the global observation angle and feature projection deformation. (**a**) Observation geometry at θ_k_ = 0°. (**b**) Observation geometry at θ_k_ = 30°. (**c**) Observation geometry at θ_k_ = 60°. (**d**) Feature projection at θ_k_ = 0°. (**e**) Feature projection at θ_k_ = 30°. (**f**) Feature projection at θ_k_ = 60°.

Let n denote the unit normal vector of the plane containing the target feature, which is obtained by fitting a plane to the edge points reconstructed using the initial equal-weight reconstruction. The optical-axis direction of the k-th camera is given by the third column of its extrinsic rotation matrix and is denoted by $z_{k}$. The angle between these two vectors is then defined as:

(20) $$\theta_{k}=\arccos\left(\left|n^{T}z_{k}\right|\right),$$

where $\theta_{k}$ denotes the angle between the normal vector of the feature plane and the camera optical axis, and is used to characterize the quality of the observation geometry for the feature under the current viewpoint. As illustrated in Figure 5, Figure 5a–c correspond to viewing angles of 0°, 30°, and 60°, respectively, while Figure 5d–f show the corresponding imaging results. When $\theta_{k}$ is small, the camera observes the feature plane approximately along its normal direction, resulting in relatively small projection distortion and better preservation of the edge geometry. As $\theta_{k}$ increases, the projected feature becomes progressively compressed and distorted, and may even undergo geometric degradation, leading to increased edge localization and 3D reconstruction errors. Therefore, $\theta_{k}$ can be used as an indicator of the overall observation quality.

To ensure that the weight decreases continuously with increasing viewing angle, an exponential decay function is adopted to define the global geometric weight:

(21) $$g_{k}=\exp\left(-\lambda_{\theta }\theta_{k}^{2}\right),$$

where $\lambda_{\theta }$ is the decay coefficient governing the rate at which the weight decreases with increasing viewing angle. Compared with a linear function, the exponential decay function maintains relatively high weights at small viewing angles, while rapidly reducing the contributions of low-quality observations once the viewing angle exceeds a certain range. This behavior is more consistent with the nonlinear increase in projection distortion with viewing angle observed in practical imaging. The coefficient $\lambda_{\theta }$ controls the decay rate of the global observation weight with increasing viewing angle. A smaller $\lambda_{\theta }$ results in a slower decay and retains relatively larger contributions from oblique observations, whereas a larger $\lambda_{\theta }$ suppresses such observations more strongly. For $\lambda_{\theta }$, changing its value does not alter the ordering of observations according to the viewing angle, but only changes the strength of their relative weighting. According to Equation (21), variations in $\lambda_{\theta }$ have a relatively limited influence at small viewing angles but become more pronounced for highly oblique observations. In the present measurement system, most valid observations have viewing angles below approximately 45°, while viewing angles greater than 60° occur only occasionally under extreme workpiece poses. With $\lambda_{\theta }=1.0$, the corresponding weights are approximately 0.76, 0.54, and 0.33 at 30°, 45°, and 60°, respectively. Therefore, $\lambda_{\theta }=1.0$ is adopted as a moderate setting that preserves useful observations within the typical viewing-angle range while progressively reducing the contribution of strongly oblique observations. According to Equation (21), when the feature-plane normal is approximately parallel to the camera optical axis, the geometric weight $g_{k}$ approaches 1. As the viewing angle increases, $g_{k}$ decreases rapidly, thereby reducing the contribution of reconstruction errors introduced by observations at large viewing angles.

It should be noted that the proposed global observation-geometry weight characterizes view-level observation quality rather than the complete geometric uncertainty of multi-view triangulation. Although camera baseline geometry and ray-intersection angles also affect reconstruction accuracy, they represent pairwise or multi-view triangulation conditions rather than the single-view observation quality considered in the present formulation. In the current measurement system, the convergent camera arrangement provides multi-directional geometric constraints and helps reduce severely unfavorable near-parallel ray configurations. However, these triangulation conditions are not explicitly modeled in the current weighting function. In addition, the feature-plane normal estimated from the initially reconstructed edge points is treated as deterministic, without explicitly propagating its estimation uncertainty into the global weight. Incorporating triangulation-conditioning information and plane-normal uncertainty propagation will be considered in future work.

### 3.3. Hybrid-Level Weight Fusion Strategy

After obtaining the local edge quality weights and the global observation-geometry weights, the proposed method performs weight fusion using each set of cross-view corresponding points as the basic unit, thereby constructing the fused weights for multi-view 3D reconstruction. For the p-th spatial edge point to be reconstructed, let its set of corresponding 2D observations across different views be denoted as:

(22) $$\left\{x_{\mathrm{pk}}|k\in V_{p}\right\},$$

where $V_{p}$ represents the set of views in which the spatial point can be observed and successfully matched. The local weight is used to evaluate the edge imaging quality and localization reliability of the same spatial point in different views, whereas the global weight evaluates the overall observation pose of the feature in the corresponding view. These two types of weights characterize observation reliability from two complementary levels, namely point-level local information and view-level global geometric information. Therefore, the final weight is constructed through a combination of hierarchical normalization and multiplicative fusion, so that the 3D reconstruction process is jointly constrained by both local edge quality and global observation geometry.

First, for the p-th set of corresponding points, let $A_{pk}$ denote the grayscale difference between the two sides of the edge in the k-th view, $\sigma_{spk}$ the grayscale transition-width parameter, and ${RMSE}_{pk}$ the root mean square error of the edge-model fitting. Let $\theta_{k}$ denote the angle between the plane normal of the feature to which the p-th spatial point belongs and the optical axis of the k-th camera. After separately normalizing the local quality weight and the global observation-geometry weight, the fused weight assigned to the 2D feature point corresponding to the p-th spatial point in the k-th image can be expressed as:

(23) $$w_{pk}=\frac{\frac{A_{pk}}{\left(\sigma_{spk}+\left.\varepsilon \right)\right.\left({RMSE}_{pk}+\left.\varepsilon \right)\right.}}{\sum_{j\in V_{p}}\frac{A_{pj}}{\left(\sigma_{spj}+\left.\varepsilon \right)\right.\left({RMSE}_{pj}+\left.\varepsilon \right)\right.}}\cdot \frac{\exp\left(-\lambda_{\theta }\theta_{pk}^{2}\right)}{\sum_{m\in V_{p}}\exp\left(-\lambda_{\theta }\theta_{pm}^{2}\right)},$$

where ε is a small positive constant introduced to prevent division by zero, and $\lambda_{\theta }$ is the decay coefficient of the global geometric weight.

Equation (23) constructs the fused weight by combining hierarchical normalization with multiplicative fusion. First, the local quality weights are normalized within the set of cross-view corresponding points associated with the current spatial point, thereby characterizing the relative localization reliability of the 2D observations across different views. Subsequently, the global geometric weights are normalized over the set of views participating in the reconstruction of that spatial point to describe the relative geometric quality of different observation poses. This hierarchical normalization preserves the physical meanings of the point-level and view-level quality evaluations, while preventing the fused weights from becoming unbalanced due to differences in weight scales across images or extreme values caused by measurement errors. It should be noted that, when the local quality weights contain extreme values due to variations in imaging quality or model-fitting errors, normalization alone may still cause the weight of an observation from a particular view to become excessively concentrated, thereby significantly weakening the effective constraints provided by corresponding observations from the remaining views. To address this issue, an upper bound is imposed on the fused weights in practical implementation. For a spatial point reconstructed from n valid cross-view observations, equal-weight reconstruction assigns a normalized weight of 1/n to each observation. Accordingly, the upper bound is set to 2∕n, allowing a high-quality observation to contribute up to twice the equal-weight contribution while preventing excessive concentration of the reconstruction weight in a single view. This choice preserves the ability of the weighting strategy to emphasize reliable observations while maintaining sufficient contributions from the remaining views to exploit multi-view geometric redundancy. Moreover, the bound automatically adapts to the number of valid observations: as n increases, the maximum contribution allowed for an individual observation decreases accordingly. The factor of 2 is not intended as a theoretically optimal value, but is adopted as a simple and interpretable compromise between emphasizing high-quality observations and maintaining multi-view geometric constraints.

To ensure that the upper-bound constraint is preserved after normalization, a bounded redistribution procedure is adopted. The fused weights are first normalized so that their sum equals one. Any weight exceeding 2∕n is then capped at 2∕n. The capped weights are kept fixed, while the remaining weight mass is redistributed among the uncapped observations in proportion to their current weights. If this redistribution causes any additional weight to exceed 2∕n, that weight is also capped and the redistribution is repeated over the remaining uncapped observations. The procedure terminates when all weights satisfy

(24) $$0\leq W_{pk}\leq \frac{2}{n},\sum_{k=1}^{n}W_{pk}=1,$$

Since the uncapped observations are scaled by the same factor during each redistribution step, their relative proportions are preserved. This procedure prevents any single observation from dominating the reconstruction while retaining the relative reliability differences among the remaining observations.

The local and global weights are fused multiplicatively because they characterize two complementary and necessary aspects of observation reliability. When the local image of an edge point is blurred, exhibits low contrast, or shows poor consistency with the fitted model, that point should not be assigned a large reconstruction weight even if the corresponding camera has a favorable global observation pose. Likewise, when a camera observes the feature at a large oblique angle, its observation may still suffer from pronounced perspective foreshortening and shape distortion, even if the local grayscale transition at the edge is relatively clear. If a weighted-sum fusion strategy were adopted, high local quality could compensate for poor global observation geometry, or favorable observation geometry could mask local edge abnormalities, causing low-reliability observations to retain relatively large weights. In contrast, multiplicative fusion imposes a joint constraint: a corresponding observation receives a high final weight only when both the local imaging quality and the global observation pose are favorable. This mechanism more effectively suppresses the influence of blurred edges, anomalous edge points, and unfavorable viewpoints on 3D reconstruction. Therefore, multiplicative fusion is more suitable for characterizing the joint reliability of multi-view observations.

To simplify the expression of Equation (23), combining Equations (17) and (21), let:

(25) $$\alpha_{pk}=\frac{q_{pk}}{\sum_{j\in V_{p}}q_{pj}},$$

(26) $$\beta_{pk}=\frac{g_{pk}}{\sum_{m\in V_{p}}g_{pm}},$$

Equation (23) can then be simplified as:

(27) $$w_{pk}=\alpha_{pk}\beta_{pk},$$

This simplified form clearly reflects the core idea of the hybrid-level weight fusion strategy presented in this section: the final weight is jointly determined by the point-level local quality weight and the view-level global geometric weight. Edge observations with reliable local localization and favorable global observation poses are assigned greater contributions to the reconstruction, whereas observations with low reliability at either level are suppressed.

In summary, the proposed method does not simply combine the local and global weights through direct addition. Instead, it adopts a hierarchical strategy consisting of local normalization within each set of cross-view corresponding points, global normalization within the set of views participating in reconstruction, and multiplicative fusion, thereby jointly evaluating observation reliability at both the point level and the view level. This strategy uses local grayscale information to characterize the quality of subpixel edge localization, while global observation geometry is employed to suppress the influence of projection distortions caused by unfavorable viewpoints. In this way, the contributions of different cross-view observations can be adaptively adjusted, error propagation can be controlled, and the accuracy, stability, and robustness of multi-view vision measurement can be improved.

## 4. Experimental Results and Discussion

The proposed hybrid-level weighted multi-view measurement method for industrial geometric features is designed to jointly evaluate the local imaging quality of cross-view corresponding points and the global observation geometry, thereby reducing the influence of low-quality edge observations and unfavorable viewpoints on 3D reconstruction and improving the accuracy, stability, and robustness of industrial geometric feature measurement. To systematically evaluate the performance of the proposed method, center-to-center distance measurement of circular holes is first adopted as the primary experimental task. Ablation experiments are conducted to compare equal-weight reconstruction, local weighting, global observation-geometry weighting, and hybrid-level weighting, so as to investigate the individual effects of the different weighting levels and their complementarity. The proposed method is then compared with existing vision-based measurement methods, and its measurement accuracy and stability are evaluated in terms of the mean error, maximum error, and standard deviation. Furthermore, random noise of different intensities is introduced into the original images to investigate the error variation and noise sensitivity of the different methods under degraded image quality, thereby evaluating their robustness to image noise. Finally, square holes, whose boundary geometry differs substantially from that of circular holes, are selected as additional measurement targets. Through 3D reconstruction of square-hole edge points and measurements of their width and height, the applicability and extensibility of the proposed method to different types of industrial geometric features are evaluated.

All experimental data were acquired using the developed multi-view measurement system. To simulate practical industrial measurement scenarios in which workpieces may be arbitrarily placed within the measurement volume, the standard workpiece was positioned at 20 different locations within the measurement region, and images were synchronously acquired by the multi-camera system at each location. Based on these experimental data, this section first introduces the multi-view measurement system, the standard workpiece, and the associated experimental conditions. Subsequently, ablation experiments on circular-hole center-to-center distance measurement, comparisons with existing methods, noise robustness experiments, and square-hole measurement experiments are conducted sequentially. The proposed method is comprehensively evaluated in terms of the effectiveness of the weighting strategy, measurement accuracy, robustness to image noise, and extensibility to different geometric features.

### 4.1. Experimental Setup

**Figure 6:**
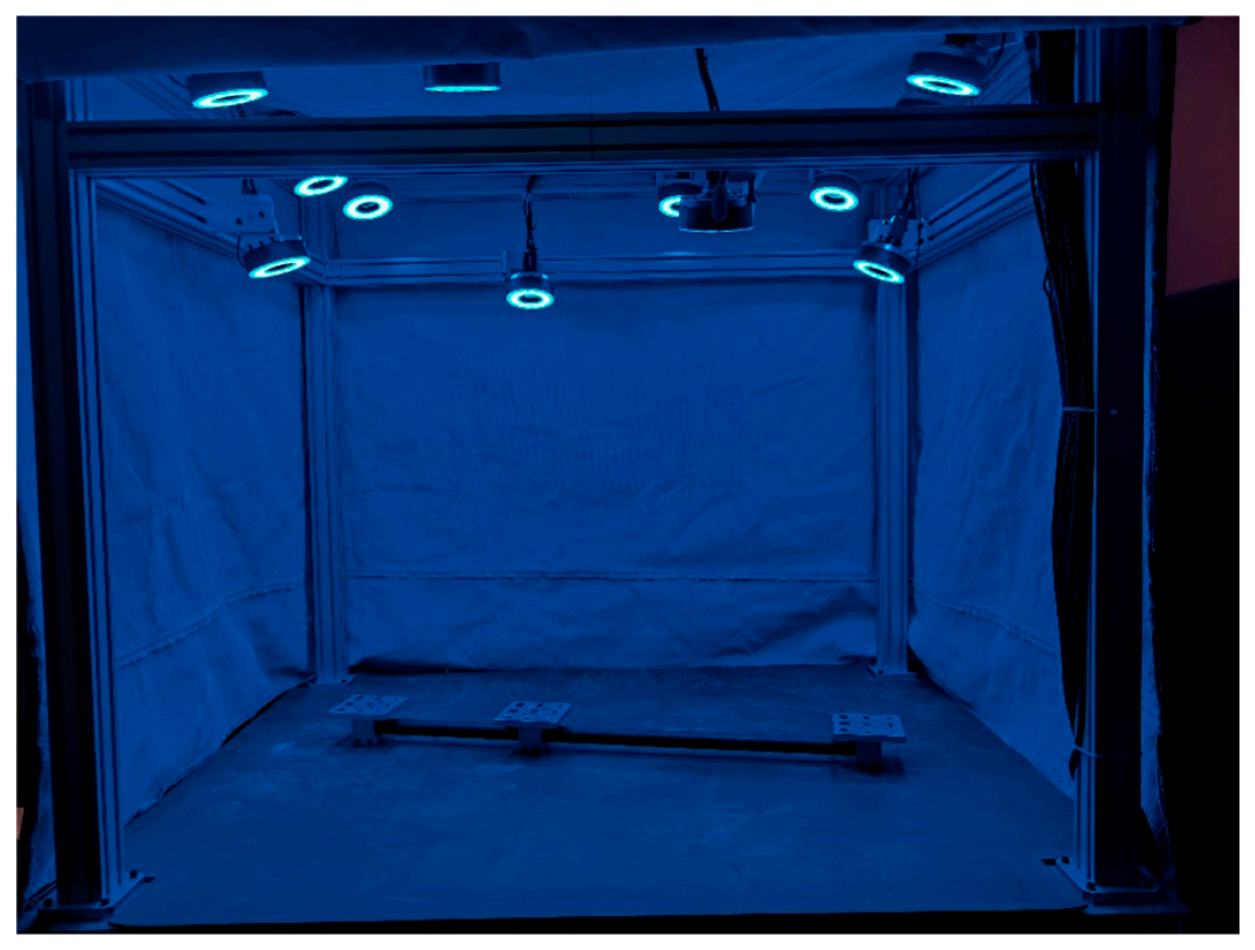
Multi-view measurement system.

The multi-view measurement system used in the experiments has a measurement volume of 1299 × 1150 × 990 mm and consists of 12 MVCA050-20GM monochrome industrial cameras (Hikrobot Technology Co., Ltd., Hangzhou, China) equipped with V1226-MPZ lenses (Computar Lenses, Tokyo, Japan). Each camera has an image resolution of 2592 × 2048 pixels and a pixel size of 4.8 um × 4.8 um. The V1226-MPZ is a fixed-focal-length industrial lens with a focal length of 12 mm. As shown in Figure 6, all cameras are mounted on a two-layer planar structure at the top of the measurement system and arranged with their optical axes converging toward the interior of the measurement volume, thereby enabling multi-view observation of the workpiece within the measurement space. Since the cameras observe the workpiece measurement region from different positions and orientations, the ground sampling distance(GSD) varies among views. The GSD of each camera was therefore evaluated at the workpiece measurement region using the calibrated camera geometry and the workpiece measurement plane. The average GSD of the 12 cameras was approximately 0.417 mm/pixel, with individual values ranging from approximately 0.359 to 0.454 mm/pixel. To reduce the influence of variations in ambient illumination during image acquisition and improve the consistency and stability of target-edge imaging, each camera is equipped with a blue ring light, and a blue optical filter is mounted in front of the lens. This imaging configuration suppresses, to some extent, interference from complex ambient illumination and enhances the grayscale contrast in the edge regions of the target features, thereby providing a stable image basis for subsequent edge extraction, feature fitting, and 3D reconstruction.

The multi-camera system was calibrated before the measurement experiments. Calibration accuracy was evaluated using the reprojection residuals of the calibration feature points. Table 1 summarizes the RMS reprojection error of each camera. The individual RMS reprojection errors ranged from 0.081 to 0.105 pixels, with an overall mean value of 0.093 pixels, indicating the calibration accuracy achieved by the multi-camera system.

To quantitatively describe the spatial configuration of the multi-camera system, the calibrated extrinsic parameters of each camera are also provided. The pose of the i-th camera with respect to the reference coordinate system is represented by the angle-axis rotation vector $r_{i}={\left[r_{i1},r_{i2},r_{i3}\right]}^{T}$ and the translation vector $t_{i}={\left[t_{i1},t_{i2},t_{i3}\right]}^{T}$. The calibrated extrinsic parameters are summarized in Table 2. These parameters completely determine the position and orientation of each camera relative to the reference coordinate system.

**Figure 7:**
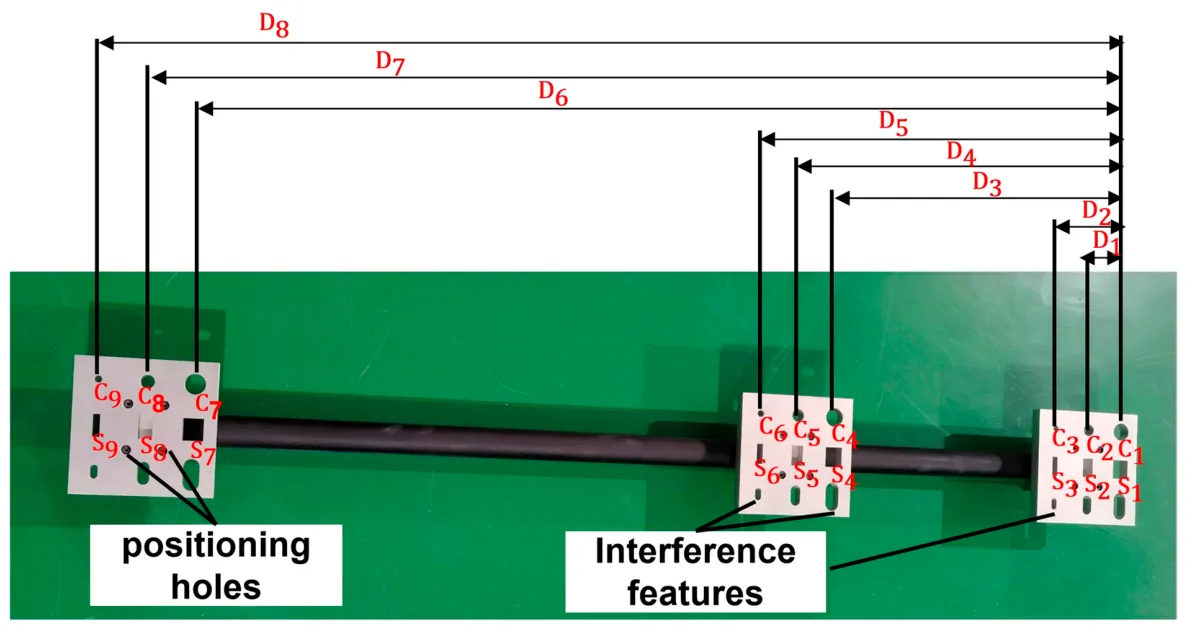
Standard workpiece.

Figure 7 shows the standard workpiece used in the experiments. The workpiece consists of three aluminum plates, each containing three circular holes, three square holes, and three U-shaped slots. The circular and square holes serve as the primary measurement targets in this study and are used to evaluate the accuracy of the proposed method in center-to-center distance measurement and square-hole dimensional measurement, respectively. The U-shaped slots are not directly measured; instead, they are introduced as interfering features to simulate the presence of complex structures and non-target features on actual industrial components, thereby creating experimental conditions that more closely resemble practical application scenarios. In addition, each aluminum plate contains four positioning holes for assembly and alignment, which are not included in the measurement evaluation.

To obtain reliable ground-truth measurements, the circular and square holes on the standard workpiece were measured in advance using a high-precision coordinate measuring machine (CMM). The CMM used in this study has a measurement accuracy of 1.6 μm, which is substantially higher than the target accuracy of the proposed multi-view measurement system. Therefore, the CMM measurements are used as the reference ground truth for the experiments. The reference values of the center-to-center distances between circular holes and the dimensions of the square holes are listed in Table 3 and Table 4, respectively. Here, C denotes the index of the circular holes, D denotes the index of the center-to-center distances, and S denotes the index of the square holes. All experiments were conducted on a laptop equipped with an Intel Core i9-13900HX CPU and 16.0 GB of RAM.

### 4.2. Ablation Experiments

To analyze the contributions of the individual components in the proposed hybrid-level weighting strategy to the measurement results, ablation experiments were conducted. Circular holes on the standard workpiece were selected as the measurement targets, and the center-to-center distance measurement error was used as the evaluation metric. Different weighting strategies were applied to circular-hole edge-point reconstruction, circle-center fitting, and center-to-center distance measurement. To ensure a fair comparison, all methods used the same camera calibration parameters, feature extraction pipeline, and 3D reconstruction framework, with only the weighting strategy being varied.

Four methods were evaluated in the ablation study: (1) equal-weight reconstruction, in which all cross-view corresponding observations are assigned identical weights; (2) local weighting, in which the proposed local strategy, including adaptive edge-position refinement and local quality weighting, is incorporated; (3) global weighting, in which only the global geometric weight is incorporated; and (4) hybrid-level weighting, which fuses the local edge quality weight and the global observation-geometry weight. By comparing these four methods, the individual effects of the local weight and global weight, as well as the effect of their fusion, on the measurement results can be systematically evaluated.

**Figure 8:**
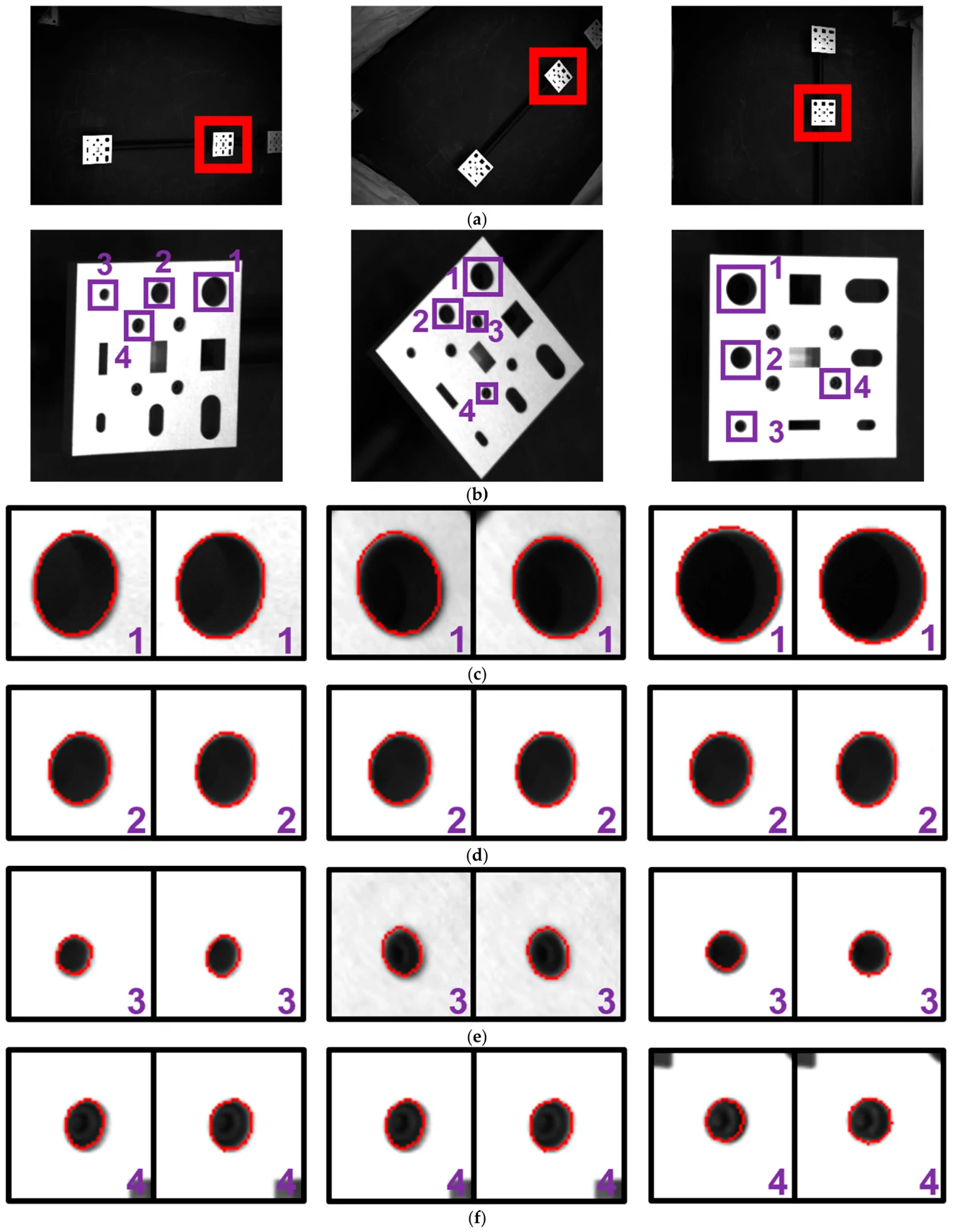
Qualitative comparison of circular-hole edge localization with and without the proposed local weighting strategy. (**a**) Undistorted images with selected regions of interest. (**b**) Enlarged views of the selected regions, with purple labels identifying the circular-hole regions used for comparison in each view. (**c**–**f**) Enlarged side-by-side comparisons for four representative circular holes. For each pair, the left image corresponds to the conventional method, and the right image corresponds to the proposed local weighting method.

To qualitatively illustrate the effect of the adaptive edge-position refinement included in the proposed local strategy, representative circular-hole images were selected to compare the conventional edge-localization method with the proposed method, as shown in Figure 8. Figure 8a shows the undistorted images and the selected local regions of interest, while Figure 8b shows enlarged views of these regions, in which the purple labels identify the four representative circular-hole regions used for comparison. Figure 8c–f provide enlarged side-by-side comparisons of the corresponding edge-localization results. In each pair, the left image shows the result obtained using the conventional method, whereas the right image shows the result obtained using the proposed local weighting method.

As can be observed from the selected regions, surface reflection, machined chamfers, local image blur, and viewpoint variations cause the circular-hole boundaries to deviate from an ideal grayscale step and instead form grayscale transition regions with finite widths. Under such imaging conditions, the conventional edge-localization method is more susceptible to local grayscale anomalies and blurred transitions, resulting in visible local fluctuations or offsets in the extracted edge contours. In contrast, the proposed local edge weighting strategy performs both adaptive edge-position refinement and reliability evaluation based on the local grayscale distribution. A subpixel edge model is first fitted along the edge-normal direction to estimate the grayscale transition center and characterize the local imaging quality. For edge regions with well-modeled grayscale transitions, the subpixel transition center obtained from the grayscale model is directly adopted as the final edge position, providing more stable subpixel localization. For other regions that may be affected by edge blur, reflection, or irregular grayscale variations, where the local grayscale information is less reliable for determining the transition center, the edge position is refined using the geometric constraint provided by the overall 2D feature fitting.

As shown in Figure 8c–f, the edge contours obtained using the proposed strategy generally exhibit smoother local variations and better consistency with the visually apparent boundary than those obtained using the conventional method. The differences are particularly evident in locally blurred or nonuniform regions, where the conventional results show relatively pronounced local offsets, whereas the proposed results follow the boundary more closely and consistently after adaptive position refinement. These qualitative observations illustrate the two complementary effects of the proposed local strategy: improving the localization of unreliable edge points through adaptive position refinement and reducing their influence on subsequent multi-view reconstruction through reliability-based weighting. Consequently, more stable edge observations are provided for subsequent feature fitting and 3D reconstruction. It should be noted that Figure 8 is intended to provide a qualitative visualization of the differences in edge localization. The quantitative effect of the proposed weighting strategy on measurement accuracy is evaluated in the subsequent ablation and comparative experiments using the CMM measurements as reference values.

**Figure 9:**
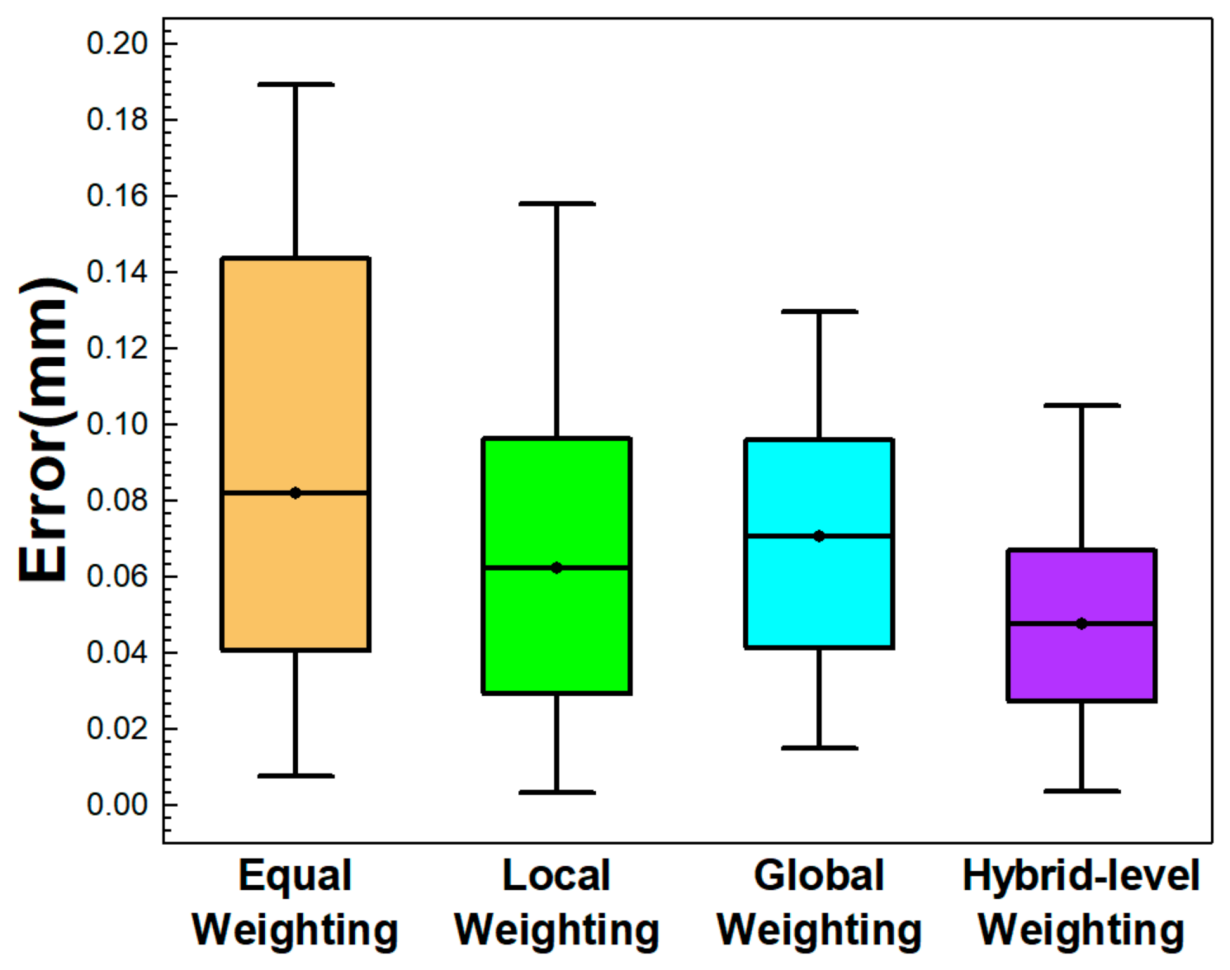
Box plots of center-to-center distance errors under different weighting strategies.

Building on the qualitative analysis, the center-to-center distance measurement errors obtained under different weighting strategies were further analyzed statistically, as shown in Figure 9. The equal-weight reconstruction method exhibits the largest interquartile range and the highest upper whisker, indicating a relatively dispersed error distribution across different workpiece placements and observation conditions. Because the equal-weight strategy does not distinguish the reliability of different cross-view observations, measurements with poor local edge quality or unfavorable observation poses contribute equally to 3D reconstruction, which can lead to larger fluctuations in the measurement results.

Compared with the equal-weight reconstruction method, the local weighting method achieves a lower mean error and a narrower overall error distribution, indicating that adjusting the contributions of corresponding observations according to local edge quality can improve center-to-center distance measurement. Because this method acts directly on point-level local observations, it reduces the influence of blurred, reflective, and poorly fitted regions on spatial point estimation, with its main advantage reflected in the reduction in the mean error. The local weighting method also suppresses the maximum error to some extent. However, since it does not explicitly account for the global observation pose, relatively large measurement deviations may still occur when certain workpiece placements result in unfavorable viewing conditions.

Compared with the equal-weight reconstruction method, the error distribution of the global weighting method is noticeably more concentrated, and the upper whisker is also substantially lower. This indicates that the global geometric weight constructed from the angle between the feature-plane normal and the camera optical axis can suppress low-quality observations under unfavorable viewing angles and reduce their contributions to 3D reconstruction. Unlike the local weighting method, the global weighting method evaluates the reliability of different camera views primarily from the perspective of view-level observation geometry. It therefore provides stronger suppression of errors associated with unfavorable observation positions and extreme viewing conditions. In the final results, the reduction in the upper whisker is more pronounced than that achieved by the local weighting method.

Among the four methods, the hybrid-level weighting method demonstrates the best overall performance. As shown in Figure 9, it not only achieves a lower mean error, but also exhibits a smaller interquartile range and a markedly shorter upper whisker, indicating that the method maintains both high measurement accuracy and good error stability. Although the mean accuracy of the hybrid-level weighting method is close to that achieved by local weighting alone, its upper whisker is further reduced, indicating that the global observation-geometry weight complements the local quality weight by compensating for its limited ability to suppress errors caused by unfavorable observation poses. These results show that local weighting primarily improves point-level edge observation quality, whereas global weighting mainly suppresses the reconstruction contributions of unfavorable viewpoints. By combining the advantages of both, the hybrid-level weighting method further reduces the maximum error while maintaining high mean measurement accuracy, thereby improving the accuracy and reliability of multi-view circular-hole measurement.

### 4.3. Comparative Experiments

To further evaluate the performance of the proposed hybrid-level weighting method in circular-hole measurement, it was compared with the equal-weight reconstruction method and existing vision-based measurement methods. The comparison methods included equal-weight reconstruction, which does not distinguish observation reliability; the geometry-based weighted multi-view method proposed by Wu et al. [23]; the high-accuracy circular-hole measurement method proposed by Tao et al. [29]; and the stereo vision measurement method proposed by Malassiotis et al. [30]. The center-to-center distances between circular holes on the standard workpiece were again used as the measurement targets, under the same dataset and experimental conditions as those described above. The mean error, maximum error, and standard deviation were selected as evaluation metrics to characterize the overall measurement accuracy, the error level under the most unfavorable condition, and the stability of the measurement results, respectively.

Table 5 presents the statistical results of center-to-center distance measurement errors for the five methods. Compared with equal-weight reconstruction, the proposed hybrid-level weighting method reduces the mean error from 0.082 mm to 0.048 mm, the maximum error from 0.190 mm to 0.131 mm, and the standard deviation from 0.056 mm to 0.033 mm, corresponding to reductions of approximately 41.5%, 31.1%, and 41.1%, respectively. These results directly demonstrate that, under the same multi-view observation conditions, adaptively adjusting the reconstruction contributions of corresponding observations according to local imaging quality and global observation geometry can effectively reduce the influence of low-reliability observations on center-to-center distance estimation. The Wu method achieves a mean error of 0.078 mm, a maximum error of 0.179 mm, and a standard deviation of 0.058 mm. Compared with this method, the proposed method reduces the corresponding errors by 38.5%, 26.8%, and 43.1%, respectively. This comparison further indicates that jointly considering point-level local edge quality and view-level observation geometry can provide more stable measurement performance than weighting based primarily on the overall projected feature geometry. The Tao method achieves a mean error of 0.053 mm, a maximum error of 0.149 mm, and a standard deviation of 0.047 mm. Compared with the Tao method, the proposed method reduces these three metrics by approximately 9.4%, 12.1%, and 29.8%, respectively. Although the two methods achieve similar mean measurement accuracy, the proposed method performs better in terms of maximum error and error dispersion, indicating that jointly exploiting point-level local quality and view-level observation geometry can further suppress error fluctuations caused by different workpiece placements and unfavorable viewpoints. For the Malassiotis method, the corresponding mean error, maximum error, and standard deviation are 0.201 mm, 0.424 mm, and 0.152 mm, respectively. Compared with the Malassiotis method, the proposed method reduces these three error metrics by approximately 76.1%, 69.1%, and 78.3%, respectively, demonstrating superior measurement accuracy and stability in complex multi-camera measurement scenarios.

It should be noted that the experimental platform, working distance, measurement range, and workpiece observation poses used in this study are not entirely consistent with the experimental conditions reported in the original studies by Wu et al., Tao et al. and Malassiotis et al. Consequently, the errors obtained in the reproduced experiments are somewhat larger than the values reported in the original studies. The dataset used in this study covers 20 different workpiece placements and includes several views with relatively large viewing angles. In addition, the measurement system has a longer working distance and a larger measurement volume, imposing more demanding requirements on both edge localization and 3D reconstruction. Therefore, comparisons conducted using the same experimental data and evaluation metrics provide a more direct assessment of the relative performance of the different methods in the multi-camera measurement scenario considered in this study.

In summary, compared with equal-weight reconstruction and the three existing methods, the proposed hybrid-level weighting method achieves lower mean error, maximum error, and standard deviation. These results indicate that the proposed method not only improves the accuracy of center-to-center distance measurement, but also enhances the stability of the measurement results across different workpiece placements and observation angles. Furthermore, these results establish a performance baseline under noise-free conditions for the subsequent robustness experiments with added image noise.

### 4.4. Noise Robustness Experiments

To evaluate the robustness of the proposed hybrid-level weighting method to image noise under degraded image quality, further noise experiments were conducted for center-to-center distance measurement. Zero-mean Gaussian noise of different intensities was added to the original undistorted images, while the subsequent procedures for feature extraction, cross-view matching, 3D reconstruction, and center-to-center distance calculation were kept unchanged for all methods. The proposed method was compared with the Tao method, the Malassiotis method, and equal-weight reconstruction to analyze the error levels of the different methods under random imaging perturbations, as well as their variations relative to the baseline results obtained without additional noise.

Let the original grayscale image acquired by the k-th camera be denoted by $I_{k}(u,v)$, and the corresponding noisy image by $I_{k}^{\prime }(u,v)$. The noise addition process can then be expressed as:

(28) $$I_{k}^{\prime }\left(u,v\right)=clip\left[I_{k}\left(u,v\right)+n_{k}\left(u,v\right),0,255\right],$$

where

(29) $$n_{k}\left(u,v\right)\sim N\left(0,\sigma^{2}\right),$$

where $n_{k}\left(u,v\right)$ denotes independent Gaussian random noise following $N\left(0,\sigma^{2}\right)$, σ is the standard deviation of the Gaussian noise, and clip(·) limits the grayscale values of the noisy image to the range [0, 255]. Six noise levels, σ = 0, 2, 4, 6, 8 and 10, were considered to simulate imaging conditions ranging from no additional noise and slight random perturbations to pronounced image degradation.

To reduce the influence of randomness in the added noise on the experimental results, each noise level was independently repeated 10 times, and center-to-center distance measurements were performed using data acquired from the standard workpiece at 20 different placements. Therefore, each method produced a total of 200 measurement results at each noise level. To ensure a fair comparison, all methods used the same noisy images, camera calibration parameters, feature-matching results, and reference ground-truth values of the workpiece; only the weighting strategy used in edge localization or 3D reconstruction was varied. The mean error, maximum error, and standard deviation were adopted as evaluation metrics to characterize the overall measurement accuracy, the error level under the most unfavorable condition, and the dispersion of the measurement results, respectively. In addition, an error ratio was introduced to quantify the relative change in each error metric with respect to the corresponding baseline under the condition of no additional noise, thereby evaluating the sensitivity of the different methods to noise perturbations.

**Figure 10:**
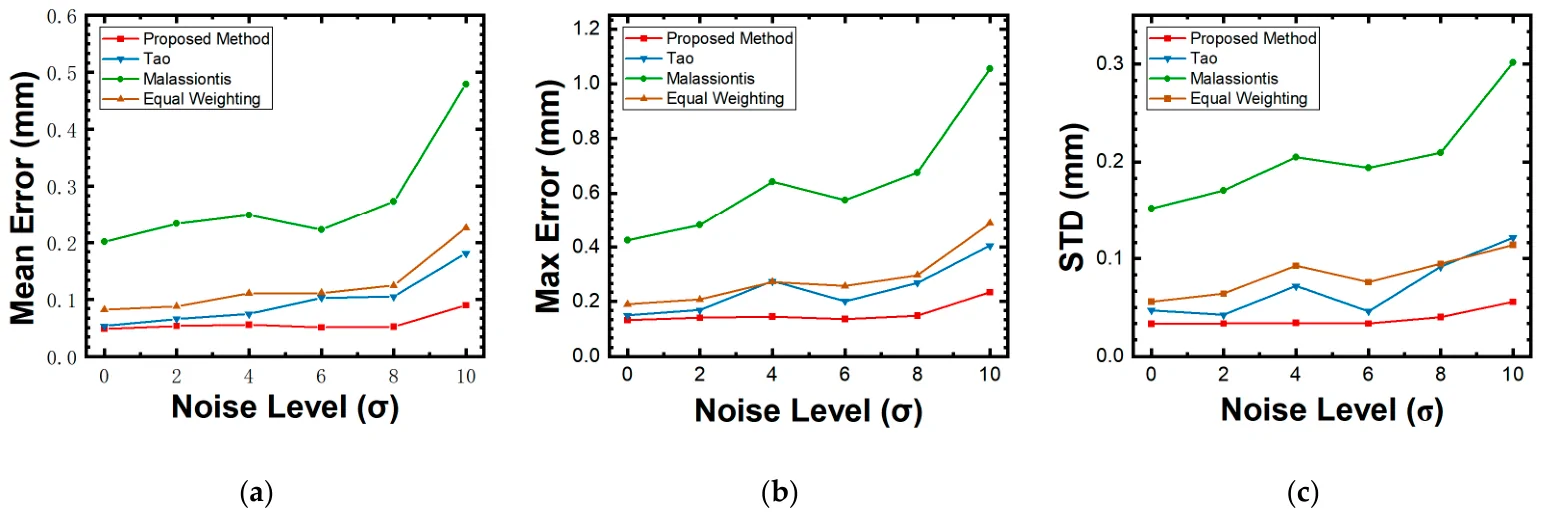
Errors of center-to-center distance measurements for different methods under different Gaussian noise levels: (**a**) mean error; (**b**) maximum error; (**c**) standard deviation.

**Figure 11:**
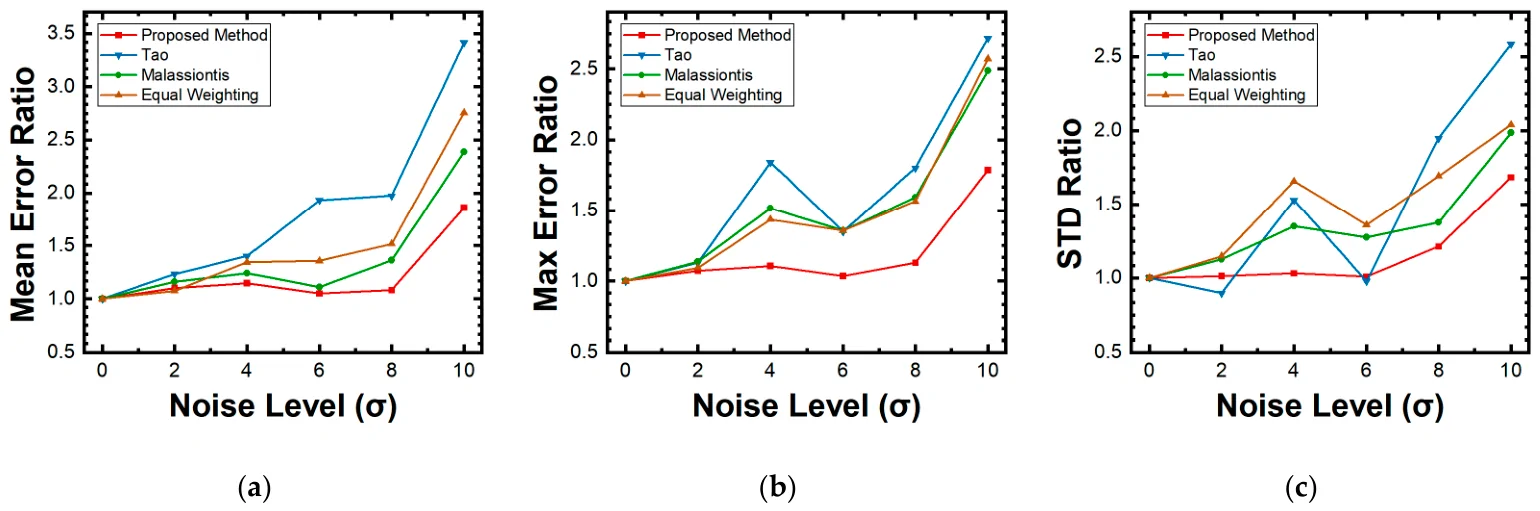
Error ratios of center-to-center distance measurements for different methods under different Gaussian noise levels: (**a**) mean error ratio; (**b**) maximum error ratio; (**c**) standard deviation ratio.

The center-to-center distance measurement results at different noise levels are shown in Figure 10 and Figure 11. Figure 10a–c present the variations in mean error, maximum error, and standard deviation with increasing noise intensity, respectively, whereas Figure 11a–c show the corresponding error ratios relative to the condition without additional noise. The error ratio is defined as:

(30) $$R_{E}\left(\sigma \right)=\frac{E\left(\sigma \right)}{E\left(0\right)},$$

where $E\left(\sigma \right)$ denotes the corresponding error metric at a noise level of σ, and $E\left(0\right)$ denotes the error metric under the condition without additional noise. The closer $R_{E}\left(\sigma \right)$ is to 1, the smaller the variation in the corresponding error metric as the noise level increases, indicating lower sensitivity of the method to noise perturbations. It should be noted that this error ratio metric primarily characterizes the relative change in error with respect to the no-additional-noise baseline and should therefore be considered together with the error metrics when evaluating the robustness of a method to image noise.

As shown in Figure 10a–c, the mean error, maximum error, and standard deviation of all four methods generally increase with increasing noise intensity, indicating that random image noise degrades edge localization accuracy and consequently affects multi-view 3D reconstruction and center-to-center distance measurement. Nevertheless, across all noise levels, the three error metrics of the proposed method remain relatively low and are overall better than those of the other comparison methods. In particular, for σ ≤ 8, the mean error, maximum error, and standard deviation of the proposed method vary only slightly, indicating that it can effectively mitigate the influence of image-quality degradation on the measurement results under low-to-moderate noise conditions. When the noise intensity increases to σ = 10, the errors of all methods increase noticeably; however, the mean error, maximum error, and standard deviation of the proposed method remain approximately 0.089 mm, 0.234 mm, and 0.056 mm, respectively, which are substantially lower than those of the other methods. In comparison, the Tao method achieves relatively low measurement errors under low-noise conditions, indicating high edge localization accuracy in this noise regime. However, as the noise intensity increases, its error metrics rise more rapidly, suggesting reduced measurement stability under stronger random perturbations. The Malassiotis method exhibits relatively large errors across different noise levels, with a particularly pronounced increase in the maximum error under high-noise conditions, indicating that its measurement accuracy is somewhat limited in the large-volume, multi-view industrial measurement scenario considered in this study. Since the equal-weight reconstruction method does not distinguish the reliability of different cross-view corresponding observations, low-quality edge observations caused by noise continue to contribute equally to 3D reconstruction, resulting in a continuous increase in measurement error as the noise intensity increases.

Because the initial measurement errors of the different methods under the no-additional-noise condition are not identical, comparison based solely on errors cannot fully reflect their sensitivity to incremental noise. Therefore, Figure 11a–c further evaluate the noise robustness of the different methods in terms of the error ratio. At σ=10, the error ratios of the mean error, maximum error, and standard deviation for the proposed method are approximately 1.859, 1.786, and 1.684, respectively, all of which are the lowest among the four methods. Moreover, at most of the remaining noise levels, the three error ratios of the proposed method also remain relatively low compared with those of the other methods, indicating that its errors increase only slightly relative to the no-additional-noise baseline as the noise intensity increases. Among the comparison methods, the Tao method exhibits relatively low error ratios in the low-noise range, indicating good stability under mild noise conditions. However, when σ>6, the error ratios of its mean error and standard deviation increase noticeably and show a more rapid upward trend in the high-noise range. This behavior may be related to its high-confidence edge selection mechanism: as the noise intensity increases, both the number and spatial coverage of valid edge points satisfying the high-confidence criterion decrease, which may weaken the geometric constraints available for subsequent ellipse fitting and consequently increase the error in circle-center estimation. The Malassiotis method exhibits relatively low error ratios at most noise levels, indicating that its measurement errors vary only gradually relative to its own no-additional-noise baseline. This may be attributed to its geometry-model-based feature estimation strategy, which provides a certain degree of robustness of the estimated feature parameters to random pixel perturbations. However, as shown in Figure 10a–c, its errors remain substantially higher than those of the other methods at all noise levels. Therefore, its relatively low error ratios only indicate a slower relative increase in measurement error and a certain degree of stability against incremental noise, rather than higher overall measurement accuracy. Since the equal-weight reconstruction method cannot adaptively adjust the reconstruction contributions of cross-view corresponding points according to changes in observation quality, its error ratios generally increase with noise intensity, indicating relatively pronounced sensitivity to noise.

The favorable noise robustness of the proposed method mainly arises from the joint evaluation of the local quality of cross-view corresponding points and the global observation geometry. As the image noise increases, random fluctuations in the grayscale distribution within the edge neighborhood become more pronounced, leading to increased fitting errors of the subpixel edge model and reduced stability of the local grayscale transition. Consequently, observations that are more strongly affected by noise are assigned lower local quality weights. Through normalization within each set of cross-view corresponding points, low-reliability observations are adaptively assigned smaller weights, whereas observations with relatively better local imaging quality receive larger weights during spatial point reconstruction. Meanwhile, the global geometric weight continues to suppress low-quality observations acquired under unfavorable observation poses, such as large viewing angles. Therefore, the proposed method jointly limits the propagation of noise-induced errors into the 3D reconstruction process from both the point-level local quality and view-level global geometry, thereby improving the stability of center-to-center distance measurement under degraded image quality.

In summary, the results of both the errors and error ratios demonstrate that the proposed hybrid-level weighting method maintains relatively low center-to-center distance measurement errors across different noise levels, while exhibiting only a limited overall increase in error relative to the no-additional-noise baseline. These results verify the stability and robustness of the proposed method against random image noise.

### 4.5. Square-Hole Measurement Experiments

To further investigate the applicability and extensibility of the proposed method to different types of planar geometric features, square-hole measurement experiments were conducted. Unlike circular holes, square-hole boundaries are mainly composed of straight edges, and their dimensional parameters are not estimated using specific geometric models such as circles or ellipses. Instead, they are obtained through 3D reconstruction of edge points, 3D line fitting, and geometric calculations between the reconstructed boundaries. Therefore, square holes differ substantially from circular holes in both boundary form and geometric parameter estimation. The square-hole measurement experiments can thus further verify whether the proposed multi-level weighting strategy can be extended from circular-hole measurement to other planar geometric feature measurement tasks based on 3D reconstruction of edge points or key points.

First, the square-hole edges were extracted from the multi-view images, and correspondences between edge points across different views were established using the calibrated camera parameters. The corresponding edge points were then reconstructed in a unified 3D coordinate system. Subsequently, the reconstructed 3D edge points associated with each boundary were fitted with spatial lines, and the three-dimensional width and height of the square holes were calculated according to the spatial geometric relationships between the fitted lines. The experiments were conducted using data acquired from the standard workpiece at 20 different placements, and the equal-weight reconstruction method was compared with the proposed hybrid-level weighting method. The mean error, maximum error, and standard deviation were adopted as evaluation metrics to characterize the overall measurement accuracy, the error level under the most unfavorable condition, and the stability of the measurement results, respectively.

**Figure 12:**
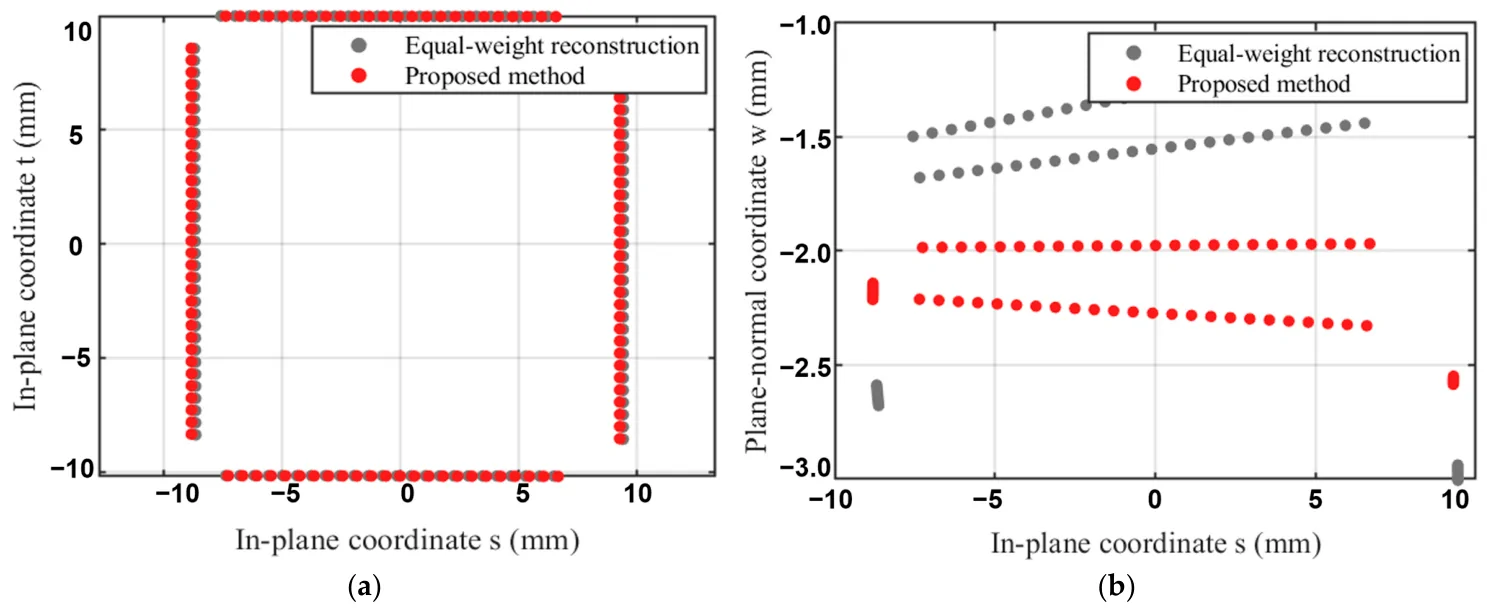
Qualitative comparison of the spatial distributions of reconstructed square-hole edge points: (**a**) top view in the measurement plane; (**b**) side view along the plane-normal direction. The red points denote the reconstructed edge points obtained using the proposed method, while the gray points denote those obtained using the equal-weight reconstruction method.

Before quantitatively analyzing the dimensional measurement errors of the square holes, the reconstructed 3D edge points were first visualized to qualitatively compare their spatial distributions under different weighting strategies, as shown in Figure 12. The red points represent the reconstructed 3D edge points obtained using the proposed method, whereas the gray points represent those obtained using equal-weight reconstruction. Figure 12a,b show the top and side views of the reconstruction results, respectively. As shown in Figure 12a, both methods can recover relatively complete square-hole contours, indicating that they are both capable of effectively reconstructing the boundary shape of the square holes within the feature plane. However, the top view mainly reflects the distribution of the reconstructed edge points within the feature plane and does not adequately reveal their dispersion along the normal direction of the feature plane. Therefore, the side view in Figure 12b is further examined to evaluate the 3D reconstruction quality of the two methods. As shown in Figure 12b, the red points reconstructed by the proposed method exhibit a more concentrated distribution and are approximately coplanar. In contrast, the gray points obtained by equal-weight reconstruction show substantially greater dispersion along the normal direction of the feature plane, with some reconstructed points distributed on both sides of the plane formed by the proposed reconstruction results. This observation indicates that, when local edge localization errors occur or imaging quality varies across viewpoints, equal-weight reconstruction cannot distinguish the reliability of different cross-view observations. Consequently, low-reliability observations contribute to the 3D estimation to the same extent as high-quality observations, making the reconstructed points more likely to deviate from the ideal feature plane and thereby reducing the coplanarity of the square-hole edge points and the accuracy of 3D reconstruction.

In contrast, the proposed method jointly evaluates the reliability of cross-view corresponding observations at two levels: point-level local edge quality and view-level global observation geometry. For low-reliability observations affected by edge blur, local grayscale anomalies, or unfavorable observation poses, the proposed method adaptively reduces their contributions to 3D reconstruction while assigning greater effective contributions to relatively high-quality observations. As a result, the reconstructed square-hole edge points exhibit better spatial dispersion and consistency, providing a more stable and reliable 3D data basis for subsequent spatial line fitting and estimation of the square-hole width and height.

To quantitatively evaluate the overall dimensional measurement performance, the width and height measurement errors obtained for the nine square holes over the different workpiece placements were statistically analyzed, as summarized in Table 6, As shown in Table 6 including square-hole width measurement results, the mean error, maximum error, and standard deviation obtained using equal-weight reconstruction are 0.101 mm, 0.240 mm, and 0.093 mm, respectively. With the proposed method, these three metrics are reduced to 0.078 mm, 0.142 mm, and 0.049 mm, corresponding to reductions of approximately 22.8%, 40.8%, and 47.3%, respectively. For square-hole height measurement, the corresponding metrics of the equal-weight reconstruction method are 0.093 mm, 0.240 mm, and 0.089 mm, whereas those of the proposed method are reduced to 0.069 mm, 0.131 mm, and 0.042 mm, corresponding to reductions of approximately 25.8%, 45.4%, and 52.8%, respectively. These results demonstrate that the proposed hybrid-level weighting method not only reduces the mean measurement errors of square-hole width and height, but also effectively decreases the dispersion of the measurement results and suppresses large deviations. This indicates that the improved spatial dispersion and consistency of the reconstructed 3D edge points can further enhance the stability of subsequent 3D line fitting and ultimately translate into improved accuracy and stability of square-hole dimensional estimation.

**Figure 13:**
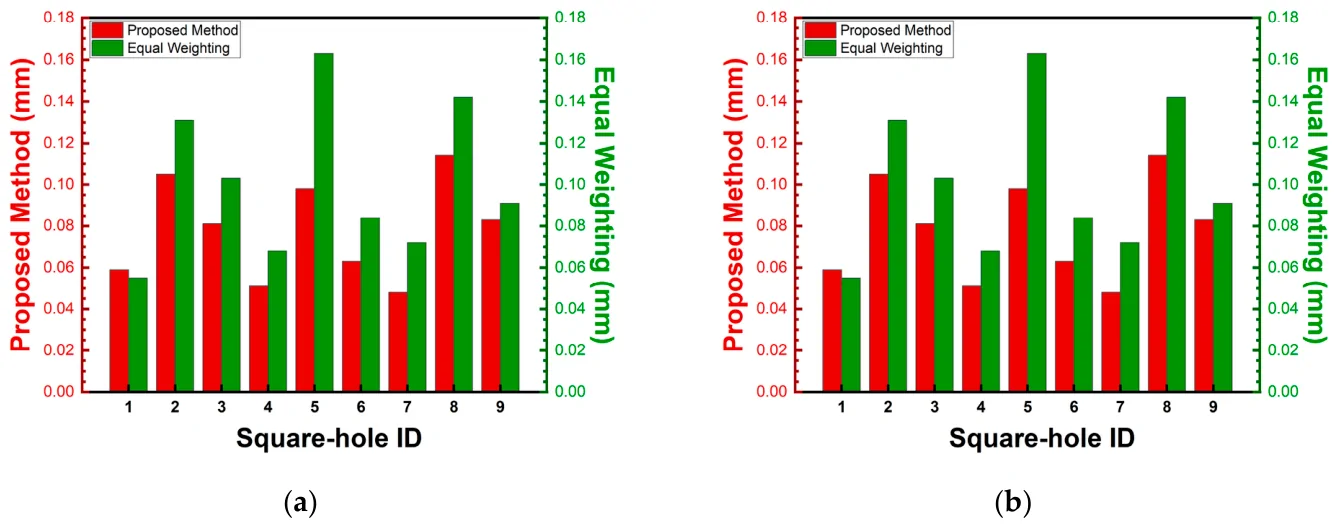
Per-hole mean measurement errors of the nine square holes: (**a**) width measurement errors; (**b**) height measurement errors.

To further examine whether the improvement was consistent across different square-hole features, the measurement errors were additionally analyzed on a per-hole basis. For each of the nine square holes, the mean width and height measurement errors over the 20 workpiece placements were calculated separately, as shown in Figure 13. Figure 13a,b present the per-hole mean errors for width and height measurements, respectively. This representation provides a more detailed view of the measurement performance at different feature locations and complements the overall statistical results reported in Table 6. As shown in Figure 13, the proposed method achieves lower mean errors than equal-weight reconstruction for most square holes in both width and height measurements. This consistent improvement indicates that the performance gain is not dominated by a particular square-hole location, but is maintained across different feature positions and observation conditions.

In summary, the square-hole measurement experiments demonstrate that the proposed hybrid-level weighted multi-view measurement method is not limited to circular features, but can also be effectively applied to square-hole measurement tasks with straight-edged boundaries. By jointly evaluating point-level local quality and view-level global observation geometry, the proposed method improves the coplanarity and spatial consistency of the reconstructed edge points, thereby further enhancing the accuracy and stability of square-hole width and height measurements. These results indicate the applicability and extensibility of the proposed method to industrial planar geometric features with different boundary forms. Although the proposed weighting framework does not explicitly depend on shape-specific projection parameters, its extension to other feature types remains subject to several limitations. The current global observation-geometry weight is based on a single feature-plane normal and is therefore mainly applicable to planar features; extension to non-planar or free-form features would require more general geometric descriptors, such as local surface normals. In addition, the local strategy relies on distinguishable grayscale transitions and reliable cross-view correspondences, and its performance may degrade under severe occlusion, weak contrast, or highly irregular edge appearances. Moreover, although the weighting framework itself is not shape-specific, the extraction of final dimensional parameters may still require feature-dependent geometric modeling.

## 5. Conclusions

To address the degradation of 3D reconstruction accuracy in industrial multi-view vision measurement caused by variations in local edge imaging quality and unfavorable observation poses, this paper proposes a hybrid-level weighted multi-view measurement method for industrial geometric features. The proposed method evaluates the reliability of cross-view corresponding observations at two levels: point-level local edge quality and view-level global observation geometry. At the local level, subpixel edge localization is achieved through grayscale modeling along the edge-normal direction, and a local quality weight is constructed by jointly considering the grayscale difference across the edge, grayscale transition width, and model-fitting error. At the global level, a global observation weight is established based on the geometric relationship between the feature-plane normal and the camera optical axis. Subsequently, each set of cross-view corresponding points is treated as the basic unit for hierarchical normalization and multiplicative fusion, and the resulting fused weights are incorporated into multi-view 3D reconstruction to reduce the propagation of errors arising from low-quality local observations and unfavorable viewpoints, thereby improving measurement accuracy and stability.

Experimental results demonstrate that the proposed method achieves high accuracy and stability in center-to-center distance measurement of circular holes. Compared with equal-weight reconstruction, the mean error, maximum error, and standard deviation are reduced by 41.5%, 31.1%, and 41.1%, respectively. In comparisons with existing methods, the proposed method also shows advantages in terms of mean error, maximum error, and standard deviation. Noise experiments further show that, as image quality degrades, both the error and error ratio of the proposed method remain relatively low overall, demonstrating good robustness to image noise. Square-hole measurement experiments further demonstrate that the proposed method improves the coplanarity and spatial consistency of reconstructed 3D edge points and reduces the mean measurement errors in width and height by 22.8% and 25.8%, respectively, validating its extensibility to different industrial planar geometric features. Future work will focus on more robust edge recognition and feature extraction methods to address feature-extraction deviations in complex through-holes caused by underlying structures, background textures, and projected internal contours, thereby further improving the applicability of multi-view vision measurement to complex industrial components.

## Figures and Tables

**Table 1:** Reprojection RMS errors of the multi-camera calibration (pixels).

Camera 1	Camera 2	Camera 3	Camera 4	Camera 5
0.091	0.101	0.083	0.081	0.105
Camera 6	Camera 7	Camera 8	Camera 9	Camera 10
0.089	0.089	0.101	0.092	0.102
Camera 11	Camera 12	Mean		
0.085	0.093	0.093		

**Table 2:** Calibrated extrinsic parameters of the multi-camera system with respect to the reference coordinate system.

Camera	$r_{1}$	$r_{2}$	$r_{3}$	$t_{1}$ (mm)	$t_{2}$ (mm)	$t_{3}$ (mm)
Camera 1	−0.276507	−0.116865	0.734002	431.675396	−520.361115	157.065548
Camera 2	−0.053492	−0.074225	−0.015636	57.863088	−338.586207	−188.961182
Camera 3	−0.006483	0.011267	−0.002137	−2.013666	3.619242	5.277553
Camera 4	0.186603	−0.046841	−0.747397	192.179737	271.795598	82.391940
Camera 5	0.349892	0.002114	−1.524846	416.209669	305.835614	−85.405503
Camera 6	0.419198	0.009306	−1.507543	347.376812	561.235868	143.106801
Camera 7	0.598996	0.015501	−2.264089	749.306718	320.740355	238.980639
Camera 8	−0.834728	−0.106298	3.020321	834.702998	−6.164992	46.307636
Camera 9	0.847720	0.097332	−3.021652	773.309306	351.359704	246.295868
Camera 10	−0.499949	−0.061161	1.496626	751.167466	−510.470500	53.308308
Camera 11	−0.460871	−0.130561	1.503595	693.624364	−262.599192	228.035293
Camera 12	−0.630115	−0.078468	2.289931	1003.210138	−36.024552	283.846516

**Table 3:** Reference center-to-center distances between circular holes measured by the CMM (mm).

Distance	D1	D2	D3	D4
Length	40.049	80.103	338.085	337.982
Distance	D5	D6	D7	D8
Length	417.912	954.263	994.227	1034.33

**Table 4:** Reference dimensions of square holes measured by the CMM (mm).

Square hole	S1	S2	S3	S4	S5
Width	20.094	20.107	20.085	20.096	20.098
Height	18.072	12.081	6.082	18.076	12.085
Square hole	S6	S7	S8	S9	
Width	20.073	20.083	20.094	20.077	
Height	6.078	18.084	12.077	6.079	

**Table 5:** Comparative results of center-to-center distance measurement.

Method	Mean Error (mm)	Max Error (mm)	Standard Deviation (mm)
Proposed method	0.048	0.131	0.033
Equal-weight reconstruction	0.082	0.190	0.056
Wu method	0.078	0.179	0.058
Tao method	0.053	0.149	0.047
Malassiotis method	0.201	0.424	0.152

**Table 6:** Comparative results of square-hole measurement.

Method	Measurement Dimension	Mean Error (mm)	Maximum Error (mm)	Standard Deviation (mm)
Equal-weight reconstruction	Width	0.101	0.240	0.093
Equal-weight reconstruction	Height	0.093	0.240	0.089
Proposed method	Width	0.078	0.142	0.049
Proposed method	Height	0.069	0.131	0.042

## Data Availability

The dataset is available on request from the author.
